# Non‐enzymatic function of WRN RECQL helicase regulates removal of topoisomerase‐I‐DNA covalent complexes and triggers NF‐κB signaling in cancer

**DOI:** 10.1111/acel.13625

**Published:** 2022-05-18

**Authors:** Pooja Gupta, Ananda Guha Majumdar, Birija Sankar Patro

**Affiliations:** ^1^ Bio‐Organic Division Bhabha Atomic Research Centre Trombay Mumbai India; ^2^ Homi Bhabha National Institute Anushaktinagar Mumbai India

**Keywords:** CHK1, NF‐κB, RECQL helicase, Topoisomerase 1, WRN

## Abstract

Mutation in Werner (WRN) RECQL helicase is associated with premature aging syndrome (Werner syndrome, WS) and predisposition to multiple cancers. In patients with solid cancers, deficiency of the WRN RECQL helicase is paradoxically associated with enhanced overall survival in response to treatment with TOP1 inhibitors, which stabilize pathological TOP1‐DNA‐covalent‐complexes (TOP1cc) on the genome. However, the underlying mechanism of WRN in development of chemoresistance to TOP1 inhibitors is not yet explored. Our whole‐genome transcriptomic analysis for ~25,000 genes showed robust activation of NF‐κB‐dependent prosurvival genes in response to TOP1cc. CRISPR‐Cas9 knockout, shRNA silencing, and under‐expression of WRN confer high‐sensitivity of multiple cancers to TOP1 inhibitor. We demonstrated that WRN orchestrates TOP1cc repair through proteasome‐dependent and proteasome‐independent process, unleashing robust ssDNA generation. This in turn ensues signal transduction for CHK1 mediated NF‐κB‐activation through IκBα‐degradation and nuclear localization of p65 protein. Intriguingly, our site‐directed mutagenesis and rescue experiments revealed that neither RECQL‐helicase nor DNA‐exonuclease enzyme activity of WRN (WRN^E84A^, WRN^K577M^, and WRN^E84A‐K577M^) were required for TOP1cc removal, ssDNA generation and signaling for NF‐κB activation. In correlation with patient data and above results, the TOP1 inhibitor‐based targeted therapy showed that WRN‐deficient melanoma tumors were highly sensitive to TOP1 inhibition in preclinical *in vivo* mouse model. Collectively, our findings identify hitherto unknown non‐enzymatic role of WRN RECQL helicase in pathological mechanisms underlying TOP1cc processing and subsequent NF‐κB‐activation, offering a potential targeted therapy for WRN‐deficient cancer patients.

## Graphical abstract

WRN RECQ helicase, an anti‐premature aging gene, acts as a critical regulator for removal of TOP1‐DNA covalent complexes, ssDNA generation and activation of CHK1 signaling for NF‐kB‐mediated chemoresistance to TOP1 inhibitors.

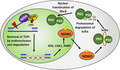

## 1 INTRODUCTION

Camptothecin (CPT) derivatives, for example, topotecan and irinotecan specifically inhibit Topoisomerase 1 (TOP1) and are clinically approved chemotherapeutics for a wide array of cancers including ovarian, colorectal and lung cancers (Thomas & Pommier, 2019). TOP1 introduces transient single‐strand breaks (SSBs) through formation of TOP1‐DNA covalent complexes (TOP1cc) and re‐joins DNA strands to allow the removal of negative as well as positive supercoiling of DNA during active replication and transcription. TOP1 inhibitors stabilize TOP1cc, leading to replication runoff and/or stalled/reversed/collapsed forks mediated DNA double‐strand breaks (DSBs) (Murai et al., 2019; Thomas & Pommier, 2019). To this end, DSB formation is primarily dependent on persistence of TOP1cc, which are lethal if not repaired (Thomas & Pommier, 2019). However, these studies have been carried out at acute micromolar concentrations of CPT, which are far higher than physiologically achievable concentrations. Understanding various molecular players involved in the removal of TOP1cc and CPT‐resistance at nanomolar concentrations may be helpful in targeting cancers with better therapeutic outcomes.

Mutation in Werner (WRN) RECQL helicase is associated with premature aging syndrome (Werner syndrome, WS) and predisposition to multiple cancers. WS patients exhibit heightened incidence of neoplasia, for example, soft tissue sarcoma, osteosarcoma, malignant melanoma, meningioma, thyroid cancer, breast cancer, and leukemias (Lauper et al., 2013; Sugimoto et al., 2011). Imperatively, it is reported that WRN expression is epigenetically downregulated in multiple cancers in patients. We and others have previously demonstrated the instrumental role of WRN in DSB repair process and its therapeutic implications in response to radiation and chemotherapy (Gupta et al., 2021; Shamanna, Lu, de Freitas, et al., 2016). It has also been shown that WS patient cells or WRN‐depleted cancer cells are hypersensitive to TOP1 inhibitors due to defective S‐phase checkpoint and repair (Cheng et al., 2008; Patro et al., 2011). Survival of colorectal cancer patients with low expression of WRN was significantly increased after TOP1 inhibitor treatment (Agrelo et al., 2006). In the METABRIC (Molecular Taxonomy of Breast Cancer International Consortium) cohort comprising 1977 breast cancers, Shamanna, Lu, Croteau et al. (2016) have shown recently that aggressiveness and adverse prognostic outcome in breast cancer patients were correlated with altered TOP1 and WRN expression in the tumor (Shamanna, Lu, Croteau, et al., 2016). Although WRN is known to remove TOP1cc through proteasome‐mediated degradation at very high (micromolar) concentrations of topotecan (Christmann et al., 2008), the precise mechanism of WRN‐mediated removal of TOP1cc and the downstream effects at physiologically relevant concentrations (nanomolar) of CPT is not yet known.

Several studies have also shown that TOP1 inhibition‐mediated DNA damage triggers NF‐κB, a transcription factor, to induce prosurvival signaling, leading to resistance in different cancers (Martin et al., 2011). In response to DSB inducing genotoxic agents, PARP1 and ATM are activated, leading to their poly ADP‐ribosylation (PARylation) and the assembly of ATM and NEMO (NF‐κB essential modifier) or IKKγ, PIASy complex (Stilmann et al., 2009). Further, NEMO is SUMOylated and phosphorylated by PIASy and ATM, respectively, to trigger its translocation to cytoplasm and activation of IκB kinase (IKK) complex (IKKα, IKKβ, and NEMO). This in turn stimulates phosphorylation, ubiquitination, and subsequent degradation of inhibitor of NF‐κB(IκBα) to release NF‐κB (p65/p50) heterodimer (Stilmann et al., 2009; Yang et al., 2011). NF‐κB (p65/p50) now freely enters the nucleus and stimulates gene expression for cell survival (Hayden & Ghosh, 2008). Since WRN‐depleted cancer cells or WS patient‐derived cells are highly sensitive to TOP1 inhibitors, we envisaged that WRN might be orchestrating TOP1cc removal and subsequent NF‐κB activation to offer therapeutic resistance in WRN‐proficient cells. Here, by using multiple approaches, we show that WRN RECQL helicase, independent of its helicase and exonuclease activity, regulates TOP1cc removal, leading to accumulation of single‐stranded DNA (ssDNA) and activation of NF‐κB to trigger robust resistance to physiologically relevant concentrations of CPT *in vitro* and *in vivo*.

## 2 RESULTS

### 2.1 WRN triggers intrinsic resistance via NF‐κB activation in response to TOP1cc

**FIGURE 1:**
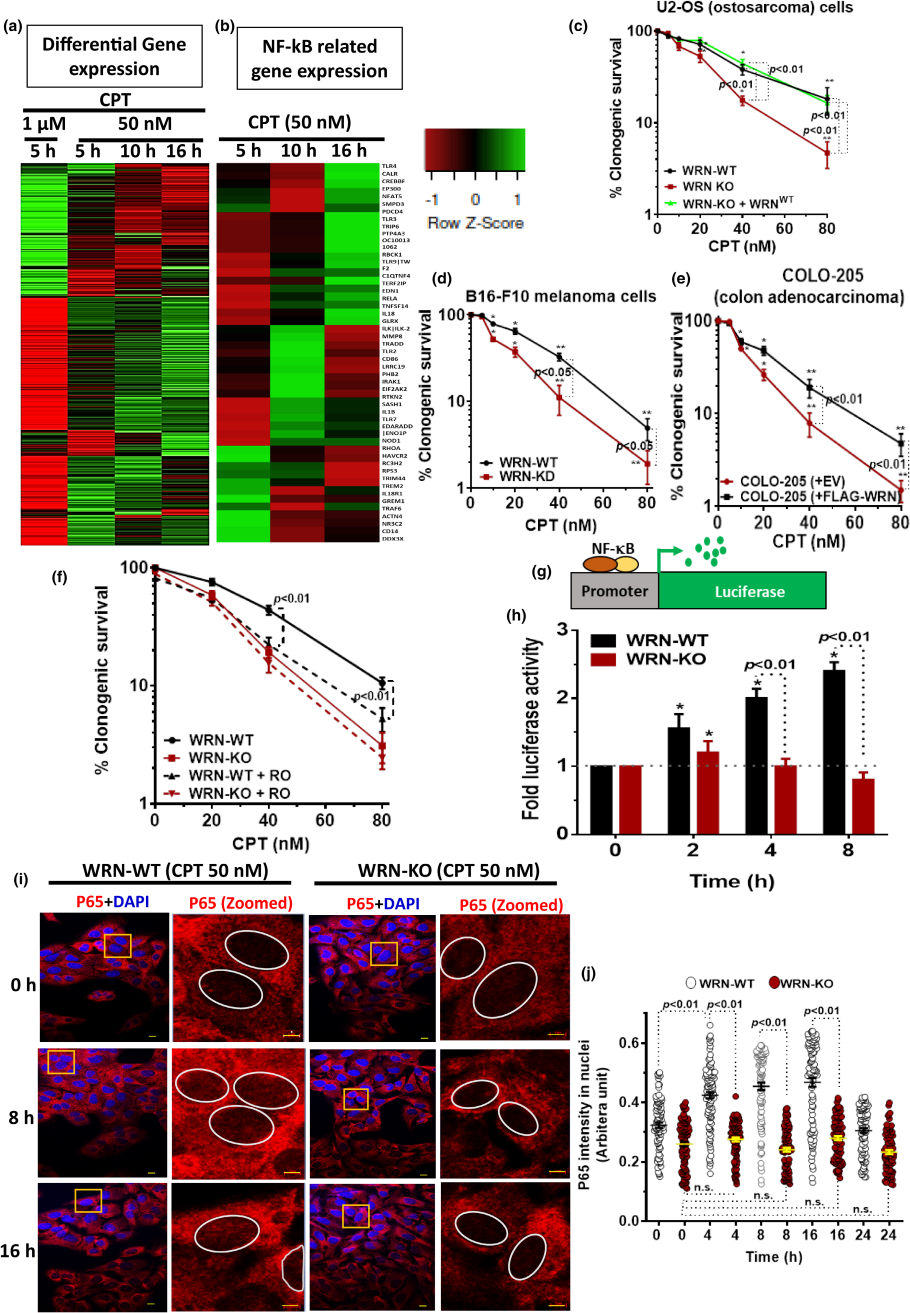
WRN regulates activation of NF‐κB gene expression and induces intrinsic chemoresistance in response to TOP1cc. (a) Microarray expression profile of overall differential gene expression (DGE) with cutoff 1.5‐fold in samples treated with 1 μM (5 h) and 50 nM (5–16 h) of CPT. (b) Heatmap of NF‐κB related DGE in response to CPT (50 nM) at different time points. (c–e) WRN‐proficient and WRN‐deficient U2‐OS, B16‐F10, and COLO‐205 cancer cells were treated with indicated concentration of CPT for 2 days and clonogenic survival of cells in drug‐free medium were assessed. (f) WRN‐WT and WRN‐KO U2‐OS cells were treated with CPT, as mentioned above, in the absence or presence of NF‐κB inhibitor (Ro 106‐9920, 1 µM) and clonogenic survival was assessed. (g) Schematic representation of luciferase expression through NF‐κB promoter (h) WRN‐WT and WRN‐KO cells expressing NF‐κB driven luciferase reporter were treated with CPT (50 nM) for indicated time periods, and NF‐κB activation was assessed in terms of fold increase in luciferase activity. (i,j) WRN‐WT and WRN‐KO cells were treated with CPT (50 nM), for indicated time periods, and NF‐κB activation was assessed in term of nuclear translocation of p65 (Bar: 10 μm; zoomed images: 5 μm). Quantification of p65 intensity in the nucleus is shown in (j). All the values indicated are mean ± *SEM* (*n* = 3–5). *p < 0.05 and **p < 0.01 with respect to respective untreated control cells. ns, not significant

Previously, we have shown that WRN expression protects osteosarcoma cells from the toxic effects of the TOP1ccs at micromolar concentration of CPT (Patro et al., 2011). In order to unravel the hitherto unknown role of WRN in CPT‐resistance in response to TOP1cc, we initially performed whole‐genome transcriptomic analysis of mRNAs in U2‐OS cells treated with nanomolar concentration of CPT. Upon analysis, we observed a drastic change in the landscape of differential gene expression (DGE) at nanomolar concentration in time‐dependent manner and also with higher concentration of CPT (i.e., micromolar) (Figure 1a). The DGE at nanomolar concentration of CPT was unexpectedly different from the DGE at micromolar concentration of CPT (Figure 1a, Table S1). Further, to annotate the pathways activated at nanomolar concentration of CPT treatment, gene ontology (GO) based pathway classification was performed using PANTHER (Protein ANalysis THrough Evolutionary Relationships) classification system (Mi et al., 2019), which revealed that NF‐κB‐regulated genes were upregulated in response to nanomolar concentration of CPT (Figure 1b, Figures S1 and S2). In order to evaluate the role of WRN in cancer resistance to TOP1cc, WRN expression was abolished in U2‐OS osteosarcoma by CRISPR‐Cas9 double nickase system (WRN‐KO vs. WRN‐WT cells expressing control CRISPR‐Cas9 double nickase vector; Figure S3a). Our results revealed that WRN‐deficient (WRN‐KO) cells showed higher sensitivity to CPT in clonogenic assay, which was rescued by ectopic expression of WRN in WRN‐KO cells (Figure 1c). We also depleted WRN in B16‐F10 melanoma cells by shRNA expressing lentiviral system or overexpressed WRN in COLO‐205 colon carcinoma cells (Figure S3b,c), which are known have low WRN expression due to epigenetic silencing (Agrelo et al., 2006). In both B16‐F10 melanoma and COLO205 colon carcinoma cells, WRN deficiency was strongly associated with enhanced sensitivity to nanomolar concentrations of CPT (Figure 1d,e). In order to unravel whether WRN expression might be linked to NF‐κB activation and *de novo* resistance to CPT treatment, we assessed the CPT sensitivity of WRN‐proficient cells (WRN‐WT U2‐OS) in the presence of an NF‐κB‐specific pharmacological inhibitor (Ro 106–9920). We found that NF‐κB inhibition significantly enhanced sensitivity of WRN‐WT cells in response to CPT while it had no or marginal effect on WRN‐KO cells, indicating a key role of WRN in NF‐κB activation mediated CPT‐resistance (Figure 1f). We further employed a luciferase‐based NF‐κB reporter assay (Figure 1g), in order to assess the role of WRN in activation of NF‐κB at nanomolar concentration of CPT. In accordance with our microarray results, we found a significant time‐dependent enhancement of luciferase (NF‐κB) activity in WRN‐WT cells, even at nanomolar concentrations of CPT, while no or marginal increase was observed in WRN‐KO cells (Figure 1h). Consequently, IκBα degradation, a critical requirement for NF‐κB activation, was rapidly and significantly enhanced in WRN‐WT cells while it was significantly abrogated in WRN‐KO cells in response to CPT treatment (Figure S3d,e). Moreover, nuclear translocation of p65 (NF‐κB), from cytoplasm, was observed with CPT treatment in a time‐dependent manner in WRN‐WT cells, which was significantly ameliorated in WRN‐KO cells (Figure 1i,j). Altogether, these results showed a novel role of WRN in triggering intrinsic/*de novo* resistance in cancers toward CPT to TOP1 inhibition through regulation of NF‐κB activation.

### 2.2 WRN regulates NF‐κB activation via CHK1 and PARP1

**FIGURE 2:**
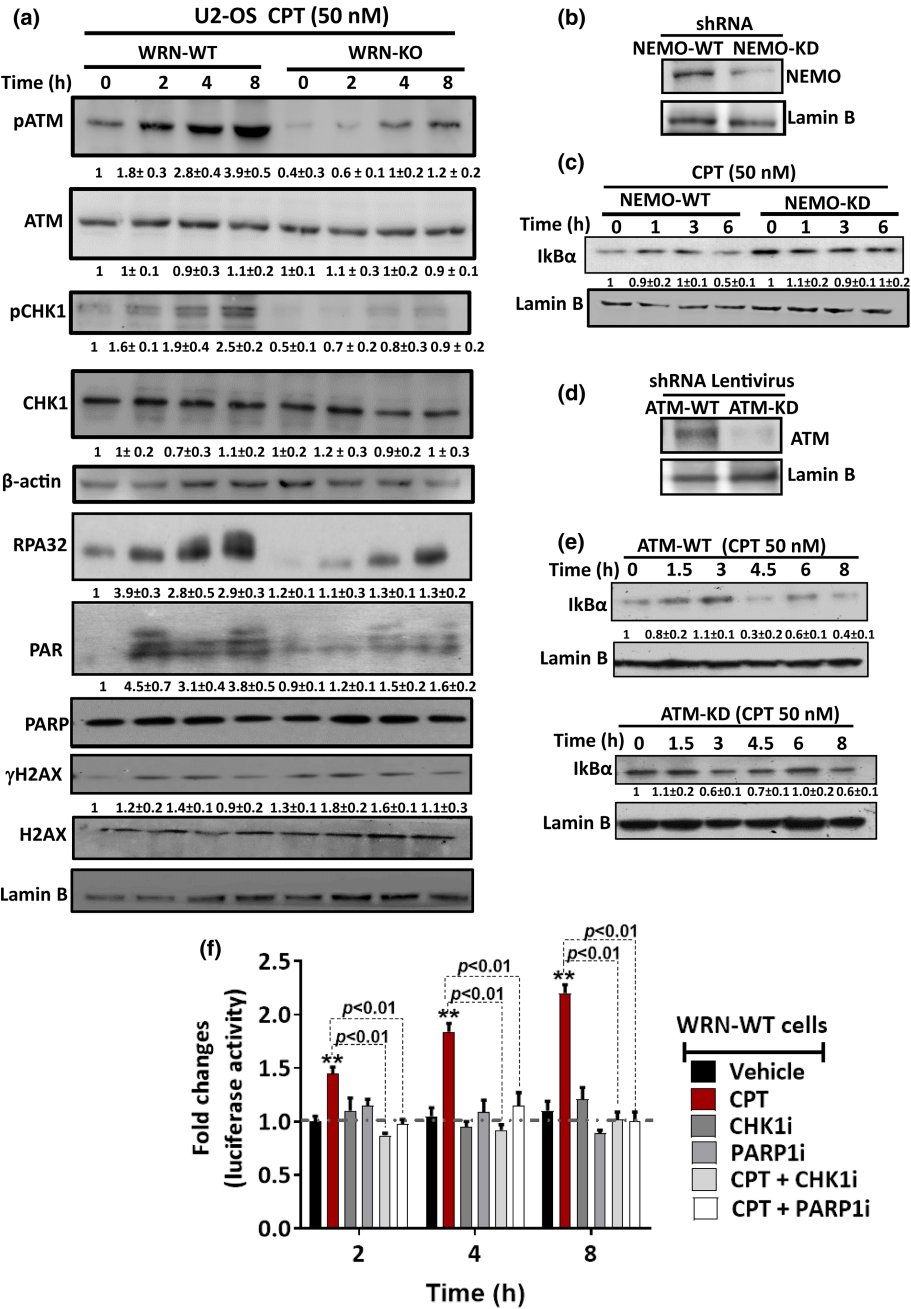
WRN‐mediated NF‐κB activation requires CHK1 and PARP1. (a) U2‐OS Cells were treated with CPT (50 nM) for indicated time periods, and activation of DDR proteins was assessed by Western blotting. (b) NEMO‐knockdown (NEMO‐KD) U2‐OS cells were generated by lentivirus mediated shRNA expression system. Expression of NEMO protein in NEMO‐WT (control shRNA) and NEMO‐KD cells was assessed by Western blotting. (c) NEMO‐WT and NEMO‐KD cells were treated with CPT (50 nM) for indicated time periods, and NF‐κB activation was assessed in terms of IκBα degradation by Western blotting. (d) ATM‐knockdown (ATM‐KD) U2‐OS cells were generated by lentivirus mediated shRNA expression system. Expression of ATM protein in ATM‐WT (control shRNA) and ATM‐KD cells was assessed by Western blotting. (e) ATM‐WT and ATM‐KD cells were treated with CPT (50 nM) for indicated time periods, and NF‐κB activation was assessed in term of IκBα degradation by Western blotting. (f) WRN‐WT cells expressing NF‐κB driven luciferase reporter were treated with CPT (50 nM) in the absence or presence of CHK1i or PARP1i for indicated time periods and NF‐κB activation was assessed in terms of fold increase in luciferase activity, which is normalized by renilla expression. All the values indicated are mean ± *SD* (*n* = 3 for a, c, e) or mean ± *SEM* (*n* = 4 for f). **p < 0.01 with respect to vehicle treatment at respective time points

Although extensive studies are available for TOP1cc formation, DNA repair and NF‐κB activation in response to micromolar concentrations of TOP1 inhibitors, only few studies describe the remodeling of replication forks in response to clinically relevant nanomolar concentrations of TOP1 inhibitors (Berti et al., 2013; Iannascoli et al., 2015; Ray Chaudhuri et al., 2012). However, the precise role of TOP1cc dynamics and NF‐κB activation at such low concentrations of TOP1 inhibitor is not known so far. To identify the WRN regulated pathway that might activate NF‐κB at nanomolar concentrations of CPT, we assessed activation of DNA damage response (DDR) proteins. Our results revealed that phosphorylation of ATM, RPA2, H2AX and CHK1, as well as PARP mediated PARylation were enhanced in a time‐dependent manner in WRN‐WT cells in response to CPT (Figure 2a). In contrast, activation of these proteins was severely downregulated/delayed in WRN‐KO cells (Figure 2a). This result was in agreement with our previous studies (Gupta et al., 2021; Patro et al., 2011), showing that WRN‐deficient cells are defective in the activation of ATM and ATR‐CHK1 signaling in response to genotoxic agents (Figure 2a). Previously, we and others have shown that WRN undergoes degradation in response to ionizing radiation (Gupta et al., 2021) and high concentration of CPT (10 μM) (Shamanna, Lu, Croteau, et al., 2016). Interestingly, WRN mRNA level was enhanced while WRN protein level remained unaffected in response to physiologically relevant nanomolar concentration of CPT (Figure S4a,b). Therefore, the above observed effects were not related to perturbed WRN protein level in WT cells. We also observed that EdU (5‐ethynyl 2'‐deoxyuridine) incorporation was similar in WRN‐WT and WRN‐KO cells (Figure S5b), suggesting the compromised DDR is also not related to differential S‐phase distribution in these cells.

Further, we sought to know whether ATM and NEMO mediated activation of canonical NF‐κB pathway, a primary mechanism in various genotoxic stimuli (Stilmann et al., 2009), might also be associated with cellular response to nanomolar concentrations of CPT. In this regard, IκBα degradation was severely abrogated in NEMO silenced (NEMO‐KD) or ATM silenced cells (ATM‐KD) in response to CPT (Figure 2b–e), indicating that canonical NF‐κB pathway activated with nanomolar concentration of CPT treatment. Since PARP1 and CHK1 activity was severely downregulated in WRN‐KO cells, their role in WRN‐mediated activation of NF‐κB pathway was assessed. Imperatively, specific inhibitors of CHK1 (SCH 900776; CHK1i) and PARP1 (BMN673, PARPi) completely abolished NF‐κB‐dependent luciferase expression in response to CPT treatment at different time points (Figure 2f). Similar results were also observed in CHK1 or PARP1 depleted cells (siRNA mediated; data not shown). Moreover, CHK1i and PARPi treatment reduced IκBα degradation and enhanced sensitivity of WRN‐WT cells in response to CPT (Figure S6a‐d), suggesting a critical role of PARP1 and CHK1 in WRN‐mediated activation of NF‐κB pathway. This observation is in line with previous reports of CHK1‐mediated activation of NF‐κB in response to replication stress (Crawley et al., 2015; Schmitt et al., 2011).

### 2.3 WRN regulates TOP1cc removal through TDP1 and CTIP

TOP1 inhibitors induce TOP1cc mediated SSBs and replication runoff for generation of DSBs (Thomas & Pommier, 2019). In order to identify the molecular damage responsible for WRN‐mediated NF‐κB activation, IκBα degradation in response to CPT was assessed in the presence of aphidicolin (APH). APH is known to block replication runoff and enhance stalled replication forks, through its ability to inhibit DNA polymerase (Seiler et al., 2007; Su et al., 2014). In this regard, our results showed that IκBα degradation was grossly unperturbed by presence of APH in response to CPT in WRN‐WT cells (Figure S7a,b), suggesting a minimal role of replication structures/progress in WRN‐mediated NF‐κB activation, in response to nanomolar concentration of CPT.

**FIGURE 3:**
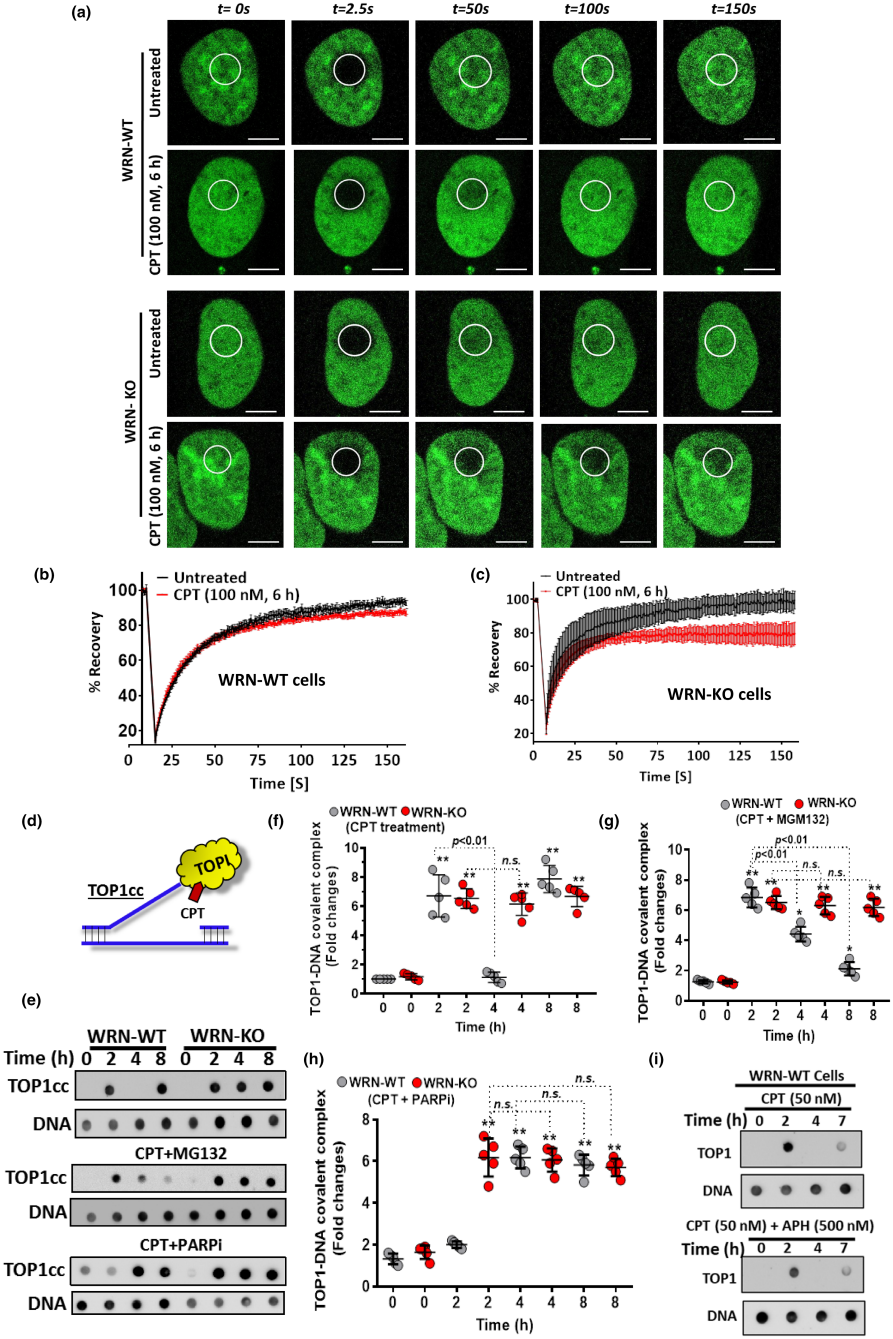
WRN is a key regulator of TOP1cc removal and NF‐κB activation. (a–c) WRN‐WT and WRN‐KO cells expressing EGFP‐TOP1 were pretreated with CPT (100 nM) for 6 h. Fluorescence of EGFP‐TOP1, at a small ROI in the nucleus, was bleached with laser irradiation, and fluorescence recovery was assessed by confocal microscopy at indicated time points (Bar: 10 μm). Quantification of fluorescence recovery is shown in B and C. Error bars represent mean ± *SE* (*n* = 10). (d) Schematic representation for stabilized TOP1cc in the presence of CPT. (e–h) WRN‐WT and WRN‐KO cells were treated with CPT (50 nM) in the absence or presence of MG132 (10 μM) or PARPi (100 nM) for indicated time periods, and TOP1cc was analyzed by RADAR based slot blot assay. DNA was probed as a loading control, in the same samples by DNA‐specific antibody. Quantification of dot blot assay is shown in F‐H. (i) WRN‐WT and WRN‐KO cells were treated with CPT (50 nM) in the absence of presence of APH (500 nM) for indicated time periods, and TOP1cc level was analyzed by RADAR based slot blot assay. All the values indicated are mean ± *SEM* (*n* = 5) **p < 0.01 with respect to vehicle treatment in the respective cell types. [Correction added on 01 June 2022, after first online publication: The blots shown in the Figure 3e were inadvertently distorted and have been corrected in this version].

Further, we focused on the role WRN in TOP1cc removal and NF‐κB activation. First, we assessed TOP1cc formation in live cancer cells by employing a previously reported confocal microscopy‐based FRAP (Fluorescence Recovery After Photobleaching) assay (Das et al., 2016). In this assay, ectopically expressing GFP‐tagged human TOP1 (EGFP‐TOP1) was photobleached at a small region of interest (ROI) in the cell nucleus and kinetics of replacement of the photobleached EGFP‐TOP1 in ROI by fluorescent EGFP‐TOP1 proteins was assessed in the absence or presence of CPT (Figure 3a). As shown in Figure 3a,b, we observed almost complete fluorescence recovery after photobleaching in both WRN‐WT and WRN‐KO cells in the absence of CPT (Figure 3a–c). [Correction added on 1 June 2022, after first online publication: Figure citations have been updated throughout the text]. This suggests that a small fraction of TOP1 participate in forming transient covalent complex with DNA (reversible TOP1cc), which can be quickly replaced by the mobile TOP1 in WRN‐WT and WRN‐KO cells. In contrast, in the presence of CPT, both the recovery kinetics (early and late) in WRN‐KO cells were significantly retarded as compared to WRN‐WT cells (Figure 3a–c). This was also reflected in the fluorescence recovery of EGFP‐TOP1 endpoint values (~10%–15% population of EGFP‐TOP1 was affected in WRN‐WT cells while 30% was affected in WRN‐KO cells) (Figure 3a–c), suggesting a key regulatory role of WRN in the removal of TOP1cc at nanomolar concentrations of CPT. To further validate the role of WRN in TOP1cc removal (Figure 3d), a slot blot‐based RADAR (rapid approach to DNA adduct recovery) assay was used (Kiianitsa & Maizels, 2013). TOP1cc formation was rapidly induced at 2 h, which was reduced at 4 h while newer TOP1cc appeared at 8 h in WRN‐WT cells in response to nanomolar concentration of CPT, indicating an intact TOP1cc dynamics (formation and removal) in WRN‐WT cells (Figure 3e,f). In contrast, although TOP1cc formation occurred normally, its removal was almost completely abrogated in WRN‐KO cells (Figure 3e,f). A known proteasome‐specific inhibitor, MG132, partially reduced/delayed TOP1cc removal in WRN‐WT cells (Figure 3e (middle panel), g). Moreover, PARPi also abrogated/delayed TOP1cc removal in WRN‐WT cells (Figure 3e (lower panel), h). In contrast, MG132 or PARPi had little impact on TOP1cc removal in WRN‐KO cells, indicating a key role of WRN in proteasome and PARP1 mediated removal of TOP1cc at low concentration of CPT (Figure 3e‐h). In a control experiment, we also found that TOP1cc removal was not affected by inhibition of replication with APH treatment in response to CPT (Figure 3i). These results verified that WRN regulates TOP1cc removal (Figure 3f), which was not grossly affected by replication structures/fork progress in proliferating cells. Further, role of WRN in TOP1cc removal in serum starved non‐proliferating cells in quiescent stage of cell cycle was assessed. As shown in Figure S8, the kinetics of TOP1cc formation, in response to nanomolar concentration of CPT, was significantly delayed in both WRN‐WT and KO quiescent cells. Interestingly, TOP1cc removal was not observed up to 8 h in both the cells. This result suggests TOP1cc formation and removal follows a WRN‐independent process in non‐proliferating but transcriptionally active quiescent (G_0_) cells. Further, to evaluate whether the role of WRN is U2‐OS cell line specific, HCT116 colorectal cancer cells were also employed. Note, WRN expression is severely downregulated in HCT116 due to epigenetically inactivation of WRN promoter (Agrelo et al., 2006). In accordance with this report, we too observed severe downregulation of WRN expression in HCT116 as compared to U2‐OS cells (Figure S9a). Further, TOP1cc removal was significantly affected in HCT116 (WRN‐deficient) cells than U2‐OS (WRN‐proficient) cells (Figure S9b,c). Interestingly, ectopic expression of WRN^WT^ rescued the phenotype of HCT116, leading to clearance of TOP1cc at 8 h (vide infra). Above results suggested that TOP1cc removal in U2‐OS and HCT116 cells are WRN dependent but not dependent on differential microsatellite (MS) nature in them, for example, MS stable U2‐OS vs MS instable HCT116 (Chan et al., 2019; Hile et al., 2013; Petitprez et al., 2013).

TOP1cc or partially degraded TOP1cc, through proteasome pathway, are known to be removed by TDP1 (Tyrosyl DNA phospodiesterase 1) (Lin et al., 2008) and/or endonucleases (XPF‐ERCC, MRE11, CTIP, MUS81, etc.) (Deng et al., 2005; Naegeli & Sugasawa, 2011; Nakamura et al., 2010; Sacho & Maizels, 2011) under different conditions. It is also known that WRN interacts/influences some of these proteins (Franchitto & Pichierri, 2004; Murfuni et al., 2013). Further, we sought to know whether WRN‐induced removal of TOP1cc is mediated through some of these proteins in response to low dose of CPT. To this end, our results showed that TDP1 silencing led to delay in TOP1cc removal while MRE11 silencing or inhibition of exonuclease activity MRE11 by mirin have marginal/no impact on the TOP1cc removal (Figure S10a–c). Interestingly, depletion of CTIP, by lentivirus (Gupta et al., 2021), led to significant inhibition of TOP1cc removal in response to CPT (Figure S11a,b). Together, our results suggested that WRN‐mediated TOP1cc removal might be influenced through TDP1 and CTIP proteins.

### 2.4 Neither helicase nor exonuclease activity of WRN is essential for TOP1cc removal

**FIGURE 4:**
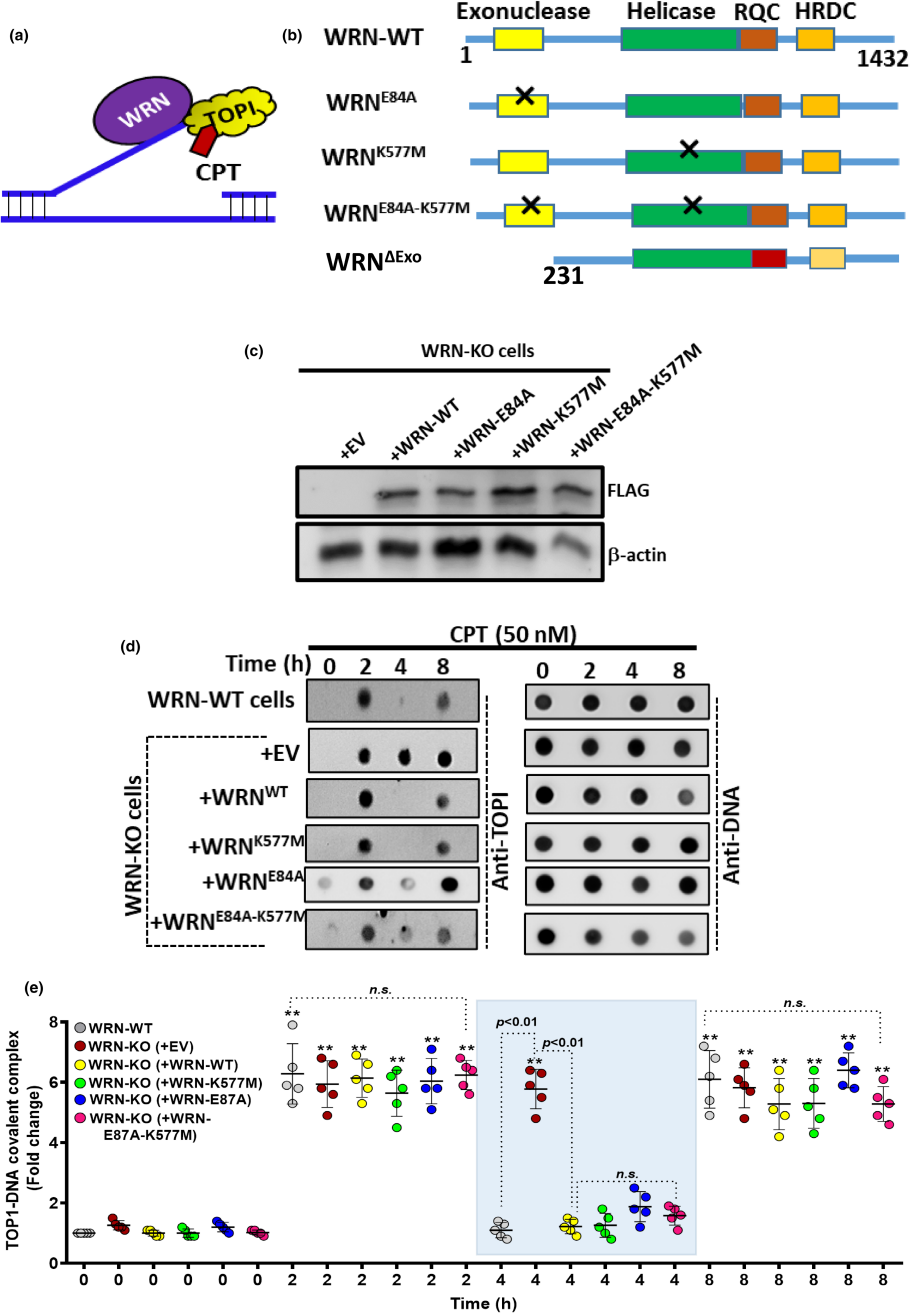
Non‐enzymatic role of WRN in TOP1cc removal. (a) Schematic representation for removal of TOP1cc by WRN. (b) Schematic showing various domains of WRN^WT^ protein. Single and double mutants of exonuclease and helicase defective WRN were also shown. (c) WRN‐KO cells were transfected for ectopic expression of EV (empty vector), WRN^WT^, WRN^E84A^, WRN^K577M^, and WRN^E84A‐K577M^. The expression level of WT and mutant WRN was assessed by Western blotting. (d, e) WRN‐KO cells were transfected for ectopic expression of EV (empty vector), WRN^WT^, WRN^E84A^, WRN^K577M^, and WRN^E84A‐K577M^. These cells were treated with CPT (50 nM) for indicated time periods, and TOP1cc was analyzed by RADAR based slot blot assay. DNA was probed as a loading control, in the same samples by DNA‐specific antibody, and all spots were quantified using densitometry. All the values indicated are mean ± *SEM* (*n* = 5) **p < 0.01 with respect to vehicle treatment in the respective cell types

WRN has exonuclease and helicase activities and multiple protein‐interacting domains and a C‐terminal motif for binding DNA (Bohr, 2008). We sought to know whether enzymatic activities of WRN are essential for TOP1cc removal at nanomolar concentration of CPT (Figure 4a). For this, FLAG‐tagged full length WRN (WRN^WT^) was mutated at 84 and 577 amino acid positions to create exonuclease (WRN^E84A^) and helicase (WRN^K577M^) defective WRN (Sharma et al., 2003), respectively, by using site‐directed mutagenesis (Figure 4b). Besides, a double mutant of WRN (WRN^E84A−K577M^), defective in both exonuclease and helicase functions, was also created (Figure 4b). WRN‐KO cells were transfected with these plasmids and were found to have similar levels of expression of WT and mutated WRN proteins (Figure 4c). As shown in Figure 4d, WRN‐KO cells (with empty vector) were defective in removal of TOP1cc *vis‐à‐vis* WRN‐WT (Figure 4d,e; please see 4 h). Ectopic expression of WRN^WT^ in WRN‐KO cells triggered complete removal of TOP1cc. Unexpectedly, ectopic expression of WRN single and double mutants, for exonuclease and helicase functions, also almost completely rescued the phenotype of WRN‐KO cells, by enabling TOP1cc repair (Figure 4d,e; please see 4 h). Earlier reports have shown the active role of exonuclease function of WRN in nascent DNA strand protection in response to CPT (Iannascoli et al., 2015). In order to further validate the redundant role of WRN exonuclease activity in TOP1cc removal, we have generated WRN^ΔExo^ deletion mutant (Figure 4b), which lacks exonuclease domain (1–230 aa) (Perry et al., 2006). Ectopic expression of WRN^WT^ and WRN^ΔExo^ almost completely rescued the phenotype of WRN‐KO cells, by enabling TOP1cc removal in response to CPT treatment (Figure S12a‐c; please see 4 h). Moreover, similar results were also observed upon complementation of WRN^WT^ and WRN^ΔExo^ in HCT116 cells in response to CPT treatment (Figure S13a,b; please see 8 h). Together, our results showed that the non‐enzymatic function of WRN regulates TOP1cc removal in response to nanomolar concentration of CPT.

### 2.5 TOP1cc removal by WRN induces ssDNA to activate CHK1 for NF‐κB activation

**FIGURE 5:**
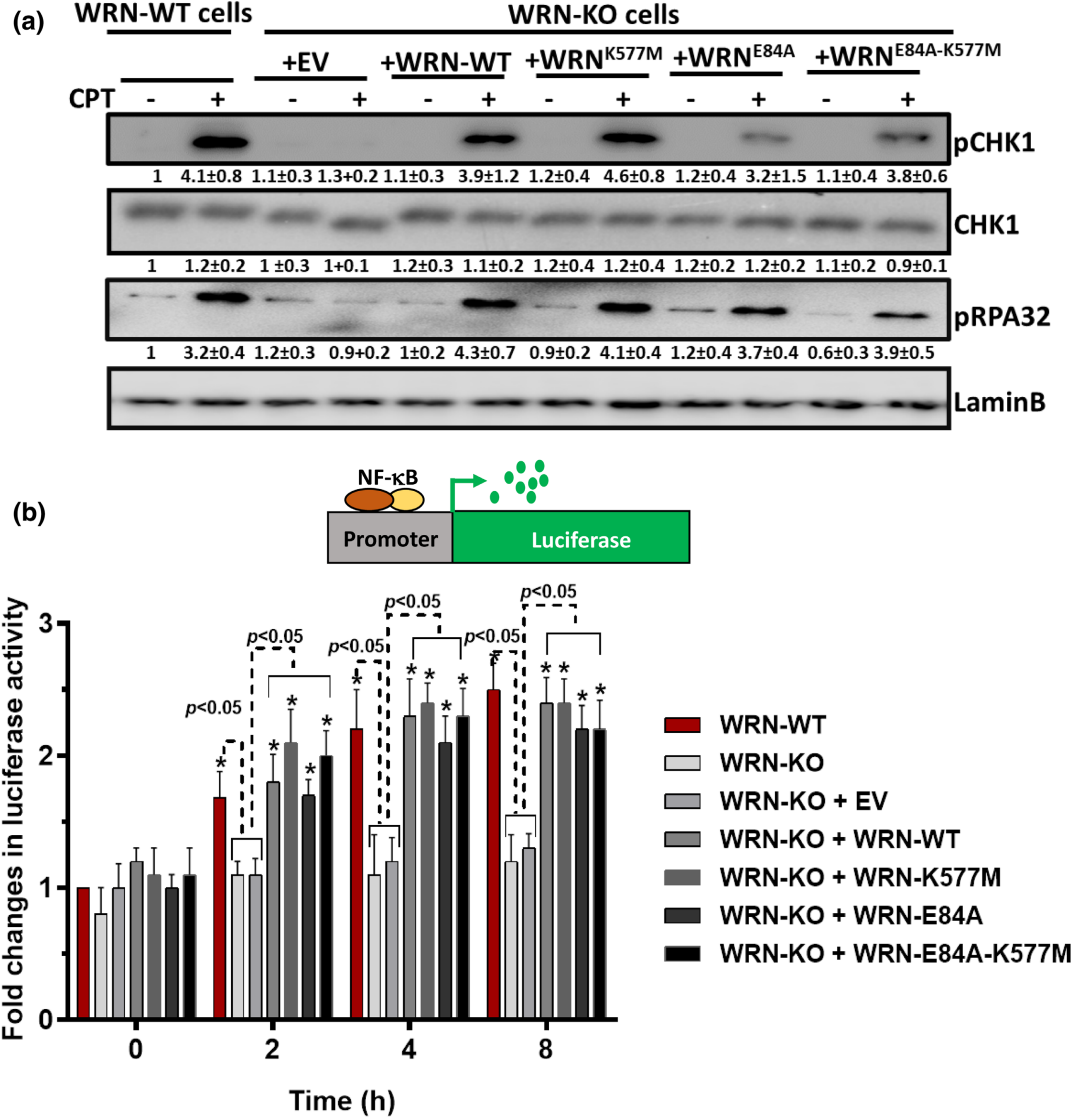
Non‐enzymatic role of WRN in ssDNA generation and activation of CHK1 and NF‐κB. (a) WRN‐KO cells were transfected for ectopic expression of EV (empty vector), WRN^WT^, WRN^E84A^, WRN^K577M^, and WRN^E84A‐K577M^. Cells were treated with CPT (50 nM) for indicated time periods, and phosphorylation of RPA2 and CHK1 was assessed by Western blotting. (b) WRN‐KO cells were transfected for ectopic expression of EV (empty vector), WRN^WT^, WRN^E84A^, WRN^K577M^, and WRN^E84A‐K577M^. Cells were treated with CPT (50 nM) for indicated time periods, and NF‐κB promoter driven luciferase expression was assessed. All the values indicated are mean ± *SEM* (*n* = 4). *p < 0.05 with respect to respective cell types at 0 h

We have previously shown that WRN induces phosphorylation of RPA2 (ssDNA formation) and CHK1 at both nanomolar (Figure 2a) and micromolar concentration of CPT (Patro et al., 2011). We also showed a key role of CHK1 in NF‐κB activation and intrinsic resistance to CPT treatment (Figure 2f and S2a,b). Next, we sought to know whether enzymatic activity of WRN is essential for ssDNA formation and CHK1 activation in response to CPT treatment. In correlation with defective TOP1cc removal, we found that both RPA2 (ssDNA) and CHK1 phosphorylation were also severely impaired in WRN‐KO cells, as compared to WRN‐WT cells in response to CPT (Figures 2a and 5a). Interestingly, ectopic expression of WRN^WT^, WRN^E84A^, WRN^K577M^, and WRN^E84A‐K577M^ in WRN‐KO cells led to significant levels of restoration in RPA2 (ssDNA) and CHK1 phosphorylation (Figure 5a). Besides, ectopic expression of WRN^WT^, WRN^E84A^, WRN^K577M^, and WRN^E84A‐K577M^ in WRN‐KO cells led to restoration of NF‐κB promoter‐based expression of luciferase (Figure 5b). Interestingly, ectopic expression of WRN^WT^ and WRN**^ΔExo^** in WRN‐KO cells also led to restoration of nuclear localization of NF‐κB (p65) in WRN‐KO cells (Figure S14a,b). In corroboration with above results, defect in TOP1cc removal, CHK1 and RPA2 phosphorylation were also observed in HCT116 (WRN‐deficient) cells. Moreover, ectopic expression of WRN^WT^ or WRN^E84A‐K577M^ rescued WRN‐deficient phenotypes (TOP1cc removal, CHK1 and RPA2 phosphorylation) of HCT116 (Figure S15). Together, these results suggest that non‐enzymatic functions of WRN are sufficient for TOP1cc removal, ssDNA formation mediated CHK1 activation and NF‐κB gene expression in response to nanomolar concentration of CPT.

**FIGURE 6:**
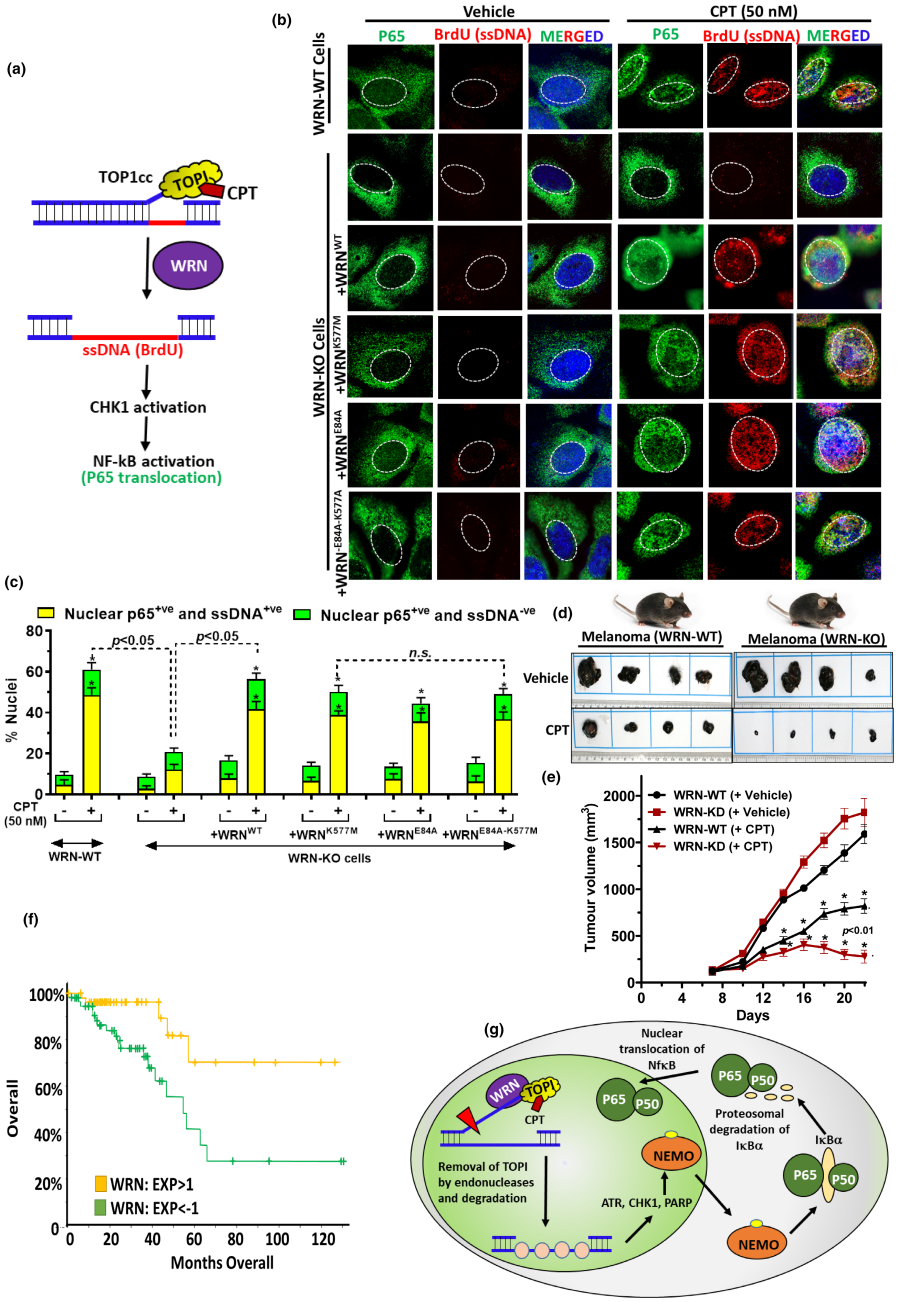
Role of WRN in ssDNA generation and nuclear translocation of p65 and its therapeutic implications. (a) Schematic representation for WRN‐mediated removal of TOP1cc, generation of ssDNA and CHK1 phosphorylation and NF‐κB activation. (b, c) WRN‐WT and WRN‐KO cells were treated with BrdU for 36 h for uniform labeling of BrdU on both the DNA strands. Further cells were exposed to CPT for indicated time periods for TOP1cc removal mediated ssDNA generation and nuclear translocation of NF‐κB (p65). BrdU exposed in ssDNA (red) and p65 (green) was assessed in non‐denaturing native condition by immunofluorescence microscopy (Bar: 5 μm). % Nuclei with more than threshold fluorescence were considered positive for p65 and/or BrdU and plotted in C. All the values indicated are mean ± *SEM* (*n* = 3). *p < 0.05 with respect to respective untreated cell types. (d,e) Mice bearing WRN‐WT and WRN‐KO melanoma tumors were treated with vehicle or CPT (1 mg/kg, once on 1st, 3rd, and 5th day per week for 4 weeks). Tumor volume was measured once every alternate day. After 30 days, mice were sacrificed and tumors were removed and analyzed. Data represent the mean ± *SD*, *n* = 6 per group. *p < 0.01 w.r.t corresponding vehicle‐treated tumor. (f) Survival of patients with WRN high and low expression in cancer using data available at cBioportal. The EXP < −1 denotes mRNA expression is <1 standard deviations (*SD*) below the mean, and EXP > 1 denotes mRNA expression is >1 *SD* above the mean. (g) Schematic model for WRN‐mediated TOP1cc removal and NF‐κB activation. In response to TOP1cc, WRN plays key role in removal of TOP1cc, leading to generation of RPA coated ssDNA and activation of CHK1 and PARP1. Subsequently, CHK1 and PARP1 may facilitate NEMO translocation to cytoplasm and activation of NF‐κB. [Correction added on 01 June 2022, after first online publication: Figure labels, 6(e) and 6(f) are misplaced and have been corrected in this version].

To obtain direct evidence for non‐enzymatic functions of WRN in ssDNA formation and subsequent activation of NF‐κB, we simultaneously assessed ssDNA and p65 nuclear translocation in single cells by immunofluorescence. Cells were allowed to grow in the presence of BrdU for 36 h, resulting in uniform BrdU incorporation in both the strands of genomic DNA. Under native non‐denaturing conditions, WRN‐mediated removal of TOP1cc exposes ssDNA (BrdU) (Figure 6a), which was assessed by immunofluorescence microscopy (Gupta et al., 2021; Patro et al., 2011). Our results revealed that CPT treatment induced ssDNA formation and a corresponding enhancement of p65 nuclear translocation in WRN‐WT cells (Figure 6b,c). In WRN‐WT cells, 48.6 ± 3.5% of nuclei were ssDNA^+ve^ and p65^+ve^ whereas in WRN‐KO cells, 12.4 ± 3.4% nuclei were ssDNA^+ve^ and p65^+ve^, suggesting a pivotal role of ssDNA in NF‐κB activation (Figure 6b,c). This further validated our previous results pertaining to the regulatory role of WRN in these two interlinked processes (Figures 1h,i and 2a). Imperatively, ectopic expression of WT, exonuclease dead, helicase dead or double mutant forms of WRN almost completely restored ssDNA formation and p65 nuclear translocation in WRN‐KO cells (Figure 6b,c). These results further validated a non‐enzymatic role of WRN in ssDNA formation and NF‐κB activation.

Altogether, these results showed that non‐enzymatic WRN plays a key role TOP1cc removal, ssDNA formation and the activation of CHK1 and NF‐κB to mediate therapeutic resistance to TOP1 inhibitors *in vitro*. Finally, we assessed therapeutic potential of TOP1 inhibitor for targeting WRN‐deficient melanoma tumor, which otherwise is known to be resistant to other therapeutic modalities (Figure 6d,e) (Gupta et al., 2021). In this regard, our results of animal studies revealed that CPT treatment (1 mg/kg body weight) significantly reduced WRN‐depleted melanoma, as compared to WRN‐proficient melanoma, indicating translational potential of TOP1 inhibitor therapy for cancer patients with WRN deficiency. Besides, we have also analyzed patient survival from TCGA database of colorectal adenocarcinoma (available at c‐Bioportal) (Cerami et al., 2012; Gao et al., 2013) and found WRN‐deficient cancer patient has more survival rate in contrast to increased WRN expressing cancer patients (Figure 6f). To this end, WRN deficiency in cancer patients may be targeted with TOP1 inhibitor therapy for better clinical outcomes.

## 3 DISCUSSION

NF‐κB plays a crucial role in chemoresistance to TOP1 inhibitors in various preclinical and clinical settings (Rasmi et al., 2020; Tomicic & Kaina, 2013). Currently, combination therapy of TOP1 inhibitors and NF‐κB inhibitors is undergoing different stages of clinical trials (Rasmi et al., 2020; Tomicic & Kaina, 2013). Although activation of NF‐κB in response to DSB inducing genotoxic stresses is known, the molecular mechanism for NF‐κB activation in response to physiologically relevant concentrations of CPT, which do not primarily cause DSBs (Volcic et al., 2012), remains elusive. To the best of our knowledge, the present study provides the first evidence that WRN RECQL protein is a key factor for activation of NF‐κB pathway, leading to intrinsic resistance to nanomolar concentrations of CPT. We have demonstrated that NF‐κB‐related genes are differentially expressed in a time‐dependent manner in response to nanomolar concentrations of CPT (Figure 1b, Table S1). Luciferase‐based reporter assay for NF‐κB activation revealed significantly higher NF‐κB activation in WRN‐WT cells compared to WRN‐KO cells in response to CPT (Figure 1g,h). In agreement with this result, IκBα degradation and subsequent translocation of NF‐κB (p65) to nucleus were significantly impeded in WRN‐KO cells, as compared to WRN‐WT cells in response to TOP1cc (Figure S3d,e and Figure 1i,j). Imperatively, WRN‐deficient osteosarcoma, melanoma, and colon carcinoma were highly sensitive to nanomolar concentration of CPT (Figure 1c–e). We also demonstrated that inhibition of NF‐κB, with Ro106‐9920, enhanced the chemosensitivity of WRN‐WT cells while the effect was marginal in WRN‐KO cancer cells (Figure 1f). Taken together, our data establish a key role of WRN in the activation of NF‐κB to induce intrinsic chemoresistance to CPT.

Replication plays an essential role in regulating sensitivity/resistance to TOP1 inhibitors, suggesting an underlying involvement of replication‐associated‐DNA‐lesions in this process. Recently, it has been shown that replication forks undergo fork reversal in response to nanomolar concentrations of CPT (Ray Chaudhuri et al., 2012). Deficiency of RECQL1 enhances the level of reversed forks in response to CPT (Berti et al., 2013). Besides, WRN is known to protect nascent replicating DNA strands from MRE11 mediated degradation at micromolar but not at nanomolar concentrations of CPT (Berti et al., 2013; Palermo et al., 2016; Su et al., 2014). At micromolar concentrations of CPT, CDK1 causes phosphorylation of WRN to induce DNA2‐dependent extensive resection of replication‐associated DSBs (collapsed replication forks). In contrast, DNA2‐mediated limited processing of nascent strands was observed in WT cells while exonuclease defective WRN causes extensive degradation of nascent strand in response to nanomolar concentrations of CPT (Iannascoli et al., 2015). It is plausible that some of these replication‐associated‐DNA‐lesions (stalled replication forks, degraded forks, reverse/regressed forks, or collapsed forks) may be responsible for activation of NF‐κB pathway in response to nanomolar concentrations of CPT. Intriguingly, our results showed that treatment with APH, a replication inhibitor (Seiler et al., 2007; Su et al., 2014), had no or marginal effects on TOP1cc formation and NF‐κB activation (Figure S7a,b and Figure 3i), suggesting replication‐associated structures/fork progress *per se* may not contribute significantly toward WRN‐mediated NF‐κB activation at lower concentration of CPT.

Accumulation of TOP1cc without repair for longer periods is known to enhance the sensitivity of proliferating cancer cells. Strategies to modify chemical structures of TOP1 inhibitors to generate stabilized and persistent TOP1cc showed enhanced sensitivity of cancer cells *in vitro* and *in vivo* (Crawley et al., 2015; Kundu et al., 2019). Persistent TOP1cc is also associated with enhanced and persistent DSBs, resulting in hypersensitivity of proliferating cancer cells (Kundu et al., 2019; Marzi et al., 2018). Faster removal of TOP1cc is considered as one of the reasons for chemoresistance to TOP1 inhibitors. Human cancer cells use both proteasome‐dependent and proteasome‐independent endonucleolytic pathways (TDP1, PARP1, XPF‐ERCC1, MRE11, CTIP, and MUS81‐EM1) to remove TOP1cc (Tomicic & Kaina, 2013). In this regard, we showed that a rapid induction of TOP1cc and its subsequent removal in WRN‐proficient cancer at nanomolar concentration of CPT, while this was abrogated in WRN‐KO cells, suggesting a key role of WRN in TOP1cc removal/repair (Figure 3a,e). Interestingly, inhibition of proteasome or PARP1 inhibitor partially or completely abrogated TOP1cc removal in WRN‐WT cells, respectively (Figure 3e). This suggested that WRN regulates TOP1cc removal through both proteasome‐dependent and independent pathways. Interestingly, TDP1 depletion delayed TOP1cc removal while CTIP depletion significantly affected TOP1cc removal, suggesting a role of WRN in influencing/recruiting TDP1 and CTIP to the site of TOP1cc (Figures S10 and S11). Imperatively, impaired helicase and/or exonuclease activities or deletion of exonuclease domain in WRN did not impede TOP1cc removal ability of WRN (Figure 4 and Figure S12), clearly suggesting a non‐enzymatic role of WRN in the process of TOP1cc removal.

Since our results revealed that replication may not play a major role in NF‐κB activation, we focused on exploring the role of TOP1cc removal/repair in this process. It is plausible that WRN‐mediated TOP1cc removal and subsequent SSB repair may involve the generation of ssDNA, which may elicit signaling to trigger NF‐κB activation. Notably, SSB repair involves DNA‐end resection by APE2 for generation of ssDNA to induce ATR‐CHK1 DDR pathway (Lin et al., 2018; Willis et al., 2013). In WRN‐WT cells, we observed RPA2 phosphorylation and ATR‐CHK1 phosphorylation in a time‐dependent manner in response to nanomolar concentrations of CPT. Phosphorylation RPA2 and CHK1 was severely defective in WRN‐KO cells (Figures 2a and 5a), showing a key role of WRN in ssDNA generation and CHK1 activation. Interestingly, helicase and exonuclease activities of WRN are not essential for these processes (Figure 5a and Figure S15). Further, the ability of non‐enzymatic WRN to resolve TOP1cc removal and ssDNA generation was significantly correlated with NF‐κB activation mediated p65 nuclear translocation at individual cell level and at cell population level (NF‐κB luciferase assay) (Figure 6a–c and Figure S14). This was defective in WRN‐KO cells in response to CPT treatment. Besides, our results revealed that WRN‐induced CHK1 activation is essential for NF‐κB activation and intrinsic resistance to CPT (Figure 2f, Figures S6a and S15). In summary, our results pinpoint WRN as a critical regulator of TOP1cc removal and ssDNA generation and activation of CHK1 signaling for NF‐κB mediated chemoresistance to TOP1 inhibitors (Figure 6g).

In conclusion, the present study establishes the significance of WRN‐mediated removal of TOP1cc and NF‐κB activation in chemoresistance of cancers to physiologically relevant concentration of CPT. Efficient removal of TOP1cc also requires PARP1 and CHK1 activation are key requirement for NF‐κB activation in response to CPT. Aggressiveness and adverse prognostic outcome in breast cancer patients was correlated with altered TOP1 and WRN expression in the tumor (Shamanna, Lu, Croteau, et al., 2016; Shamanna, Lu, de Freitas, et al., 2016). Therefore, our current investigation also provides insights to establish a foundation to devise new synthetic lethal strategies by combining CHK1 or PARP1 inhibitors with TOP1 inhibitors to counteract WRN and NF‐κB mediated intrinsic tumor resistance in clinical settings. Besides, WRN expression is downregulated in multiple cancers in patients due to epigenetic silencing. Thus, identifying WRN deficiency in individual cancer patient may help in achieving better and predictable prognosis through TOP1 inhibitor‐based personalized/targeted therapy. Our investigation, which demonstrates the association between impaired removal of TOP1cc and defective NF‐κB activation and WRN deficiency, explains enhanced survival of colorectal cancer patients with WRN deficiency in response TOP1 inhibitor treatment (Agrelo et al., 2006).

## 4 MATERIALS AND METHODS

### 4.1 Materials

Antibodies against TOP1, IκBα, phospho‐ATM, γH2AX, DNA. FLAG, β‐actin, Anti‐IKKγ/NEMO TDP1 (#SAB1411073), and H2AX were purchased from Sigma (St. Louis, MO). Antibodies against RPA2 and phospho‐RPA2 were from Bethyl Laboratories (Montgomery, TX). Antibodies against p65, WRN (#SC5629), ATM (#SC23921), CHK1 (#SC8404), Lamin B (#SC6216), and CRISPR‐Cas9 double nickase plasmid (control and WRN) were from Santa Cruz biotechnology (Santa Cruz, CA). Anti‐phospho‐345‐CHK1 was from Epitomics (Cambridge, UK). Anti‐BrdU (#347580), anti‐PAR from BD Biosciences (San Jose, CA). Anti MRE11 (#4847) from Cell Signaling Technology (Massachusetts, USA). AlexaFlour‐546 and AlexaFlour‐488 tagged secondary antibodies from Jackson ImmunoResearch, (PA, USA). Lipofectamine 2000/3000, Alexa Flour‐488/555/595 (#A21123/#A31570), prolong anti‐fade Gold was obtained from (Life Technologies, Carlsbad, CA). SCH900776 (CHK1 inhibitor) was purchased from Selleckchem (Houston, USA). Talazoparib (BMN673; PARP inhibitor) was procured from ApexBio, USA. All other reagents like Ro106‐9920, mirin (MRE11 inhibitor), camptothecin, EdU, etc., were obtained from Sigma Chemicals (St. Louis, MO), unless mentioned in the respective places.

### 4.2 Cell culture

U2‐OS cell line was purchased from American Type Culture Collection, (VA, USA) while HCT116, COLO‐205, and B16‐F10 cell lines were purchased from Sigma‐European Collection of Authenticated Cell Cultures. Cells were grown in Dulbecco's Modified Eagle's Medium (DMEM) supplemented with 10% fetal bovine serum (FBS), 2 mM glutamine, 100 U/mL penicillin, 100 μg/ml streptomycin, and 0.25 μg/ml amphotericin B in a humidified 5% CO_2_ atmosphere at 37°C. All cell lines were used for experiments within a maximum of 8 passages after thawing from the freeze vial. Cells were negative for mycoplasma throughout the current study. All the cell lines used were certified tested and authenticated by DNA profiling for polymorphic short tandem repeat markers.

### 4.3 Clonogenic assay

Cells were seeded (500/well) in 6‐well plates and were grown in the presence of vehicle or different concentrations of CPT for 24 h and then allowed to grow in CPT‐free media for 10–14 days. For combination treatment with different inhibitors, the treatment with inhibitor was given 0.5 h before CPT treatment and then removed after 24 h and allowed to grow in complete medium for 10–14 days. Subsequently, cells were washed in PBS, fixed in methanol, and stained with crystal violet (0.5% in PBS). Colonies of ≥30 cells were manually counted, and the survival curves were derived from the colony numbers normalized to respective control. COLO‐205 cells were also treated as mentioned above. After 3 days of recovery, viable cells were counted by staining with trypan blue (0.4% in PBS).

### 4.4 Generation of knockout and knockdown cells

U2‐OS cells were transfected with WRN/CRISPR‐Cas9 and control CRISPR‐Cas9 double nickase plasmid system from SCBT according to the manufacturer's protocol. Cells were selected with puromycin for stable knockout (KO) of WRN. To generate stable WRN knockdown cells, the B16‐F10 cells were exposed to lentiviral particles encoding either scrambled or WRN‐specific shRNAs (#sc‐36844‐V, Santa Cruz Biotechnology, Santa Cruz, CA) using polybrene. After 72 h of lentiviral exposure, cells were grown for two weeks in a medium containing puromycin (1 μg/ml). WRN expression in puromycin‐resistant cells was analyzed by Western blotting. Cells expressing scrambled shRNA or WRN‐shRNA are designated as WRN‐WT and WRN‐KD cells, respectively. Similarly, ATM and NEMO were depleted in U2‐OS cells, using lentiviral particles (#sc‐29363‐V, #sc‐29761‐V, Santa Cruz Biotechnology, Santa Cruz, CA), and respective clones are designated as ATM‐KD cells. For NEMO knockdown, U2‐OS cells were transfected with plasmids expressing shRNA (control or NEMO) and antibiotic resistance cells were selected. Cells expressing control and NEMO shRNA were named as NEMO‐WT and NEMO‐KD cells. CTIP‐KD cells were generated in previous report (Gupta et al., 2021). For transient depletion of TDP1 and MRE11, ON‐TARGET plus siRNA (Cat. No. J‐016112‐08‐0010 and Cat no. L‐009271‐00‐0020, respectively, from Dharmacon™) were used with lipofectamine 3000 (as per the manufacturer's protocol) for 24 h, followed by evaluation of TDP1 and MRE11 expression by Western blotting.

### 4.5 Site‐directed mutagenesis and over‐expression of WRN

To generate helicase dead, exonuclease dead or double mutated WRN, pCMV6 plasmid containing N‐terminal FLAG‐WRN (Origene, US) were used. All the mutations were generated using “QuikChange‐II” site‐directed mutagenesis kit (Stratagene, CA, USA) as per manufacturer's protocol and the mutations were confirmed by DNA sequencing. For ectopic expression of WRN, U2‐OS, HCT116, and COLO‐205 cells were transfected with pCMV6 plasmids harboring N‐terminal FLAG‐WRN (WT, K577M, E84A, or both mutations or ΔEXO) or empty vector (EV) plasmid by using lipofectamine 3000 (as per the manufacturer's protocol) for 24 h followed by evaluation of WRN expression by Western blotting. Following primers used for generating aforementioned mutations:

K577M‐Hel forward‐5′‐GATATGGAATGAGTTTGTGCTTCCAGTATCC‐3′

K577M‐Hel reverse‐5′‐CACAAACTCATTCCATATCCAGTTGCC‐3′

E84A‐Exo forward‐5′‐TGACATGGCGTGGCCACCATTATACAATAG‐3′

E84A‐Exo reverse‐5′‐GGTGGCCACGCCATGTCAAATCCCACCAC‐3′

WRN exonuclease domain (1–230) (Perry et al., 2006) was deleted by amplifying amino acid 231–1432 using primer (F_1‐⁠230 5′ATGCGATCGCCGATGATACTGTGCAAAGG 3′ and R 5′ CGTACGCGTACTAAAAAGACC 3′) and digested with restriction enzyme, AsiSI and MluI (from NEB) and ligated in pCMV‐FLAG vector. The clone plasmid with deletion was confirmed by DNA sequencing and denoted as WRN^ΔEXO^.

### 4.6 Real‐time PCR for WRN mRNA expression

Isolation of total RNA was performed using TRIzol reagent (Ambion, Life technologies). Two microgram of total RNA from each sample was used to generate cDNA by using TOPscript™ cDNA Synthesis Kit (Cat. No. EZ005M, Life technology). WRN‐specific primers (RT_F 5′AGCCACTGCCAATGGTTCCAA3′ and RT_R 5′ TCATGCCCGCAATGGTATGTT 3′) were used to amplify in real‐time reaction and monitored using SYBR green (from Biorad) in CFX96 Touch Real‐Time PCR System (Biorad, US). PCR conditions used were as follows: initial incubation at 95°C for 5 min, 40 cycles of 95°C for 12 s, 62°C for 30 s, and then one melting curve cycle. The Ct values obtained for the WRN gene were normalized to control gene GAPDH expression (Primer used for GADPH are F 5′ TCAAGGCTGAGAACGGGAAG 3′ and R 5′ CGCCCCACTTGATTTTGGAG 3′).

### 4.7 Microarray data analysis

The RNA isolation, labeling, and hybridization were conducted by a commercial Affymetrix array service (M/S Vimta Lab Services, India). The manufacturer's protocol was followed for the determination of gene expression data using Affymetrix Human GeneCHIP 1.0 ST arrays. Briefly, this method includes first and second strand cDNA synthesis, double‐stranded cDNA purification, cDNA synthesis, biotin‐labeled cDNA quantification, and cDNA fragmentation followed by subsequent hybridization. Following hybridization and washing, the Affymetrix arrays were scanned with an Affymetrix GeneChip. Image generation and feature extraction were performed using the Affymetrix Software. The data from arrays that passed the manufacturer's quality specifications were used for further analysis. The *p* value cutoff for differentially expressed genes was set at 0.05. In analysis, for differential gene analysis cutoff set for fold change is set to be log2. The heatmap of differentially gene expression (DGEs) was generated using the Heatmapper Server http://www.heatmapper.ca/expression/. The Protein ANalysis THrough Evolutionary Relationships (PANTHER) Classification System and analysis tools were used to categorize DGEs by PANTHER protein class, Gene Ontology (GO) Molecular Function, and GO Biological Process. The PANTHER Overrepresentation Test (release 20200728) was used to search the data against the GO database (Released 2020–08–10) to identify either protein classes or GO annotations overrepresented in our data when compared to a reference human genome. *p*‐values were adjusted using FDR correction.

### 4.8 NF‐κB luciferase assay

The NF‐κB luciferase reporter kit (#N1111) and the phRL‐SV40 Vector for Renilla luciferase [Cat.# E6261] were purchased from Promega. U2‐OS cells (WRN‐WT and WRN‐KO) cells were plated overnight in 96‐well plate. Cells were transfected using 0.25 μl Lipofectamine 2000 per well, with 15 ng of luciferase experimental reporter plasmid along with Renilla luciferase as internal control plasmid. After 24 h of transfection, cells were treated with vehicle/CPT in presence and absence of indicated specific pharmacological inhibitors. The plate was assayed with Dual‐Glo Luciferase Assay System (Promega, E2920) and read using a Microplate Illuminometer (BMG Labtech, POLARstar Omega). Firefly luciferase readings were normalized to Renilla luciferase readings in each well.

### 4.9 EdU labeling and staining for cell cycle

The cell cycle was assessed by incorporation of EdU in cells using Click‐iT^®^ EdU Imaging Kits (Cat no. C10337, Invitrogen). EdU (10 µM) was added to the culture media for 30 min. For staining, cells were fixed with 100% methanol and then with 1% formaldehyde for 10 min. The cells were permeabilized with 0.1% Triton‐X 100 in PBS (phosphate buffer saline) for 10 min and then blocked by 2% BSA for 1 h. The Click‐IT reactions were performed as per manufacturer's protocol. Slides were mounted in Prolong Gold anti‐fade reagent with DAPI. Images were captured using an LSM780 Meta laser scanning confocal microscope (Zeiss, Germany).

### 4.10 Slot blot assay for trapped TOP1cc

TOP1cc (TOP1‐DNA covalent complex) formation was assessed by RADAR (rapid approach to DNA adduct recovery) assay, as reported previously (Kiianitsa & Maizels, 2013). Cells were treated with CPT or vehicle and then lysed in RADAR assay lysis buffer (10 mM Tris‐Cl pH 6.8, 20 mM EDTA, 4% Triton X‐100, 6 M guanidinium isothiocyanate, 1% sarkosyl, and 60 mM DTT). The TOP1‐DNA complex were ethanol‐precipitated, washed three times in 75% (vol/vol) ethanol, and then resuspended in 8 mM NaOH. The amount of DNA obtained was quantified by Nano‐drop spectrophotometer, and equal amount of DNA were slot‐blotted onto nitrocellulose membranes and then probed with TOP1 antibodies. For loading control, DNA was probed with anti‐DNA antibody (Sigma‐Aldrich, St. Louis, MO). For starvation experiment, cells were grown in DMEM with 0.5% FBS to generate serum starvation condition for 72 h.

### 4.11 Western blotting

Cells were lysed with TNN buffer (20 mM Tris pH 7.4, 250 mM NaCl, 0.05% NP‐40) supplemented with appropriate protease and phosphatase inhibitor cocktails as mentioned previously (Patro et al., 2011). Equal amount of protein was separated by SDS‐PAGE gel and then immunoblotted with specific antibodies. Protein amounts (arbitrary unit, mean ± *SD*) were quantified by density scanning and normalized by considering that of untreated/vehicle‐treated sample as 1.

### 4.12 Fluorescence recovery after photobleaching (FRAP) assay

FRAP assay was carried out as per the reported protocol (Das et al., 2016) with minor modifications. Briefly, WRN‐WT and WRN KO U2‐OS cells stably expressing EGFP‐TOP1 (gifted by Dr. Benu Brata Das, IACS, Kolkata, India) were seeded in 35 mm thin bottom plates (2 ⅹ 10^5^ cells per plate). After 16 h, cells were treated with CPT (100 nM) for 6 h, followed by image acquisition under a Zeiss LSM780 confocal microscope. Images were acquired at 40× magnification with 6× digital zoom, ensuring pixel dwell time of 1 μs. Five pre‐bleach images were captured (488 nm laser line, 1% power) followed by bleaching of the region of interest (bleach ROI) using 20 iterations of the 488 nm laser line at 100% power. A reference region of interest was also used throughout image acquisition in order to account for photobleaching. Post bleaching 250 images were captured at 1 ms interval each. Following image acquisition, percent immobile and mobile fraction of EGFP‐TOP1 was calculated using Zen software (2.3 SP1 FP1 package). For calculation of percentage fluorescence recovery, the following formula was used:

$$\%\text{Recovery}\;\text{at}\;\text{time}\;t=\left[\frac{\left\{\frac{\text{MFI}\;\text{of}\;\text{bleach}\;\text{ROI}}{\text{MFI}\;\text{of}\;\text{reference}\;\text{ROI}}\right\}\text{at}\;\text{time}\;t}{\left\{\frac{\text{MFI}\;\text{of}\;\text{bleach}\;\text{ROI}}{\text{MFI}\;\text{of}\;\text{reference}\;\text{ROI}}\right\}\text{at}\;\text{time}\;0}\right]\times 100$$

where MFI represents mean fluorescence intensity. Recovery graphs were plotted using percentage recovery data.

### 4.13 Immunofluorescence assay

Immunofluorescence assay was carried out as per the reported protocol (Gupta et al., 2021) with minor modifications. For measuring ssDNA and p65, cells were grown on coverslips in presence of BrdU (10 μM) for 36 h, chased for 3 h in BrdU free medium, and treated with CPT for indicated time period. Subsequently, cells were fixed with 4% PFA, washed twice in PBS, and fixed with chilled methanol for overnight. For immunostaining, cells were permeabilized in Triton X‐100 (0.5% in PBS) on ice for 10 min, washed three times with PBS, and subjected to indirect immunofluorescence using primary antibody against p65 and BrdU and suitable secondary antibody tagged with Alexa‐595 and Alexa‐488. Slides were mounted in Prolong Gold anti‐fade reagent with DAPI. Images were captured using an LSM780 Meta laser scanning confocal microscope (Zeiss, Germany).

### 4.14 Animal studies

Six‐week‐old male C57BL6 mice were procured from BARC animal house facility, Mumbai, India, in accordance with ethical clearance from the BARC Animal Ethics Committee. Melanoma tumor development, housing and routine care of animals were carried out as per the standard animal maintenance guidelines and our previous report (Gupta et al., 2021). For melanoma tumor induction, B16‐F10 WRN‐WT and WRN‐KD cells (1 × 10^5^ cells/0.2 ml DMEM/mouse) were injected subcutaneously in the right flank of the mice. Tumor‐bearing mice were randomly grouped (6 mice/group), and tumors were allowed to grow to an average volume of 100 mm^3^ before treatment. Palpable tumors were noticed approximately after 7 days of tumor induction. Mice bearing palpable melanoma tumors were treated with vehicle or CPT (1 mg/kg in three doses on 1st, 3rd, and 5th day per week) through i.p. injection. During treatment, tumor volumes were measured by caliper once every alternate day and calculated according to the formula (L × W^2^)/2 (L, length; W, width). Upon completion of the experiments, the mice were sacrificed after an overdose of thiopental, the tumors were excised and their weights measured. The animals were dissected, and macroscopic analysis was also carried out to visualize major morphological changes in the organs.

### 4.15 Statistical analysis

The data are presented as the mean ± *SEM* or mean ± *SD*. Comparisons between two groups were performed using an unpaired Student's *t*‐test in GraphPad software. Comparisons between two groups were performed using one‐way analysis of variance (ANOVA) and two‐way ANOVA for multiple comparisons in GraphPad software. A value of *p* < 0.05 was considered statistically significant.

## CONFLICT OF INTEREST

None declared.

## AUTHOR CONTRIBUTIONS

BSP and PG involved in conceptualization. PG, AGM, and BSP involved in investigation, methodology, data analysis, writing original draft and revised manuscript. BSP involved in supervision.

## Supporting information

Supplementary Material

## Data Availability

All data are available in the main text or the supplementary materials. Additional data related to this paper may be requested from the authors.
