# Supplementary material for: Non‐enzymatic function of WRN RECQL helicase regulates removal of topoisomerase‐I‐DNA covalent complexes and triggers NF‐κB signaling in cancer
Source: Aging Cell. 2022 May 18;21(6):e13625. doi: 10.1111/acel.13625 (PMC9197415; doi:10.1111/acel.13625)
Supplement: Supplementary file 1 — Supplementary Material [file ACEL-21-e13625-s001.docx]

Supplementary Materials for

**Non-enzymatic function of WRN RECQL helicase regulates removal of topoisomerase-I-DNA covalent complexes and triggers NF-κB signaling in cancer**

*WRN regulates TOP1cc removal and NF-κB activation*

Pooja Gupta,^1,2^ Ananda Guha Majumdar,^1,2^ Birija Sankar Patro ^1,2^*

Correspondence to: Birija Sankar Patro, [bisank@barc.gov.in](mailto:bisank@barc.gov.in)

**Supplementary Figure 1-15. DGE for NF-kB related genes**

**Supplementary Table 1. DGE for NF-kB related genes**


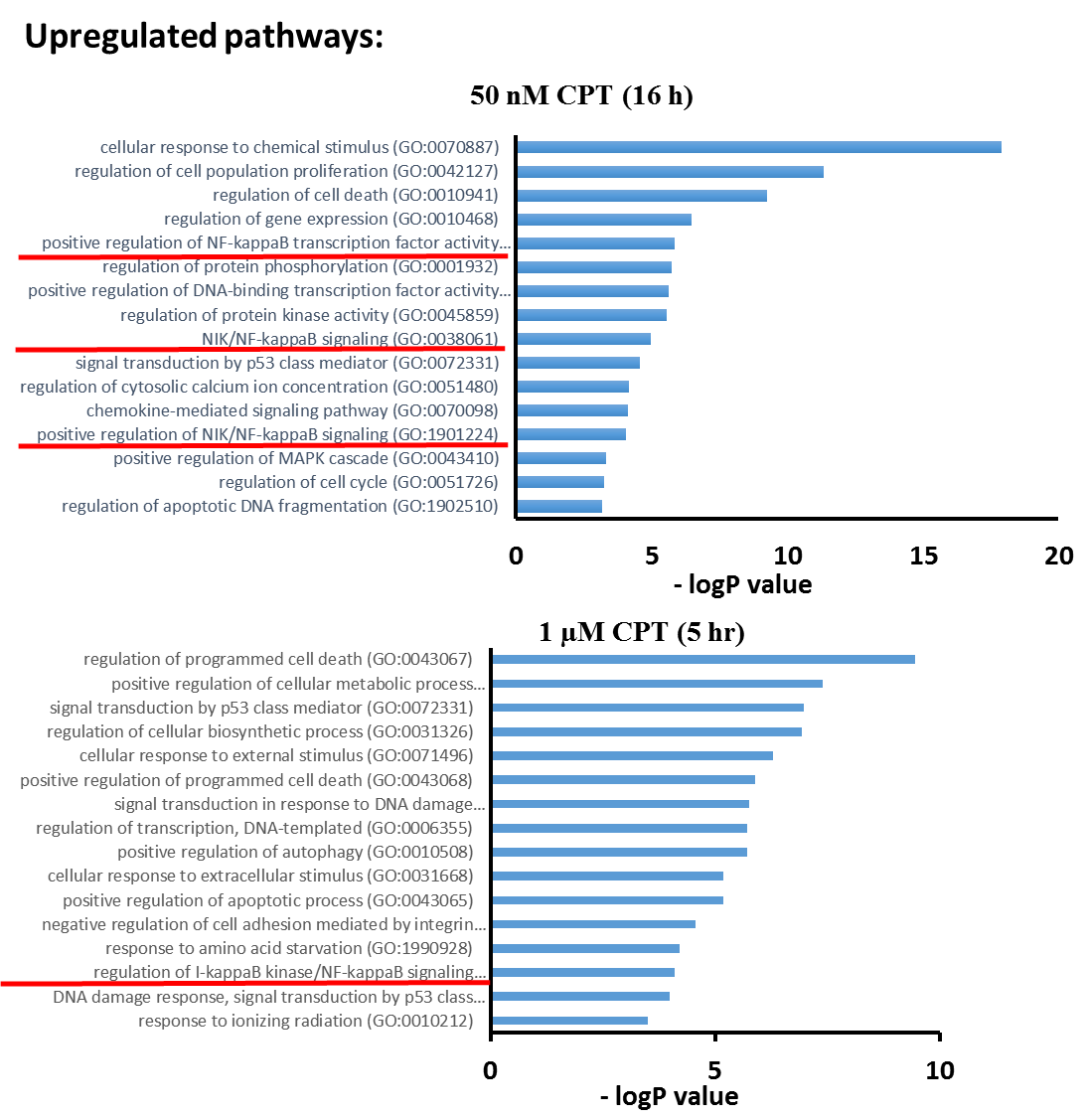


Supplementary Figure 1. Upregulated pathways in response to nanomolar and micromolar concentration of CPT. Pathway analysis are carried out from the DGE in high-throughput microarray data, obtained from cancer cells treated with 1 μM (5 h) and 50 nM (16 h) of CPT. NF-κB pathway upregulation were highlighted.


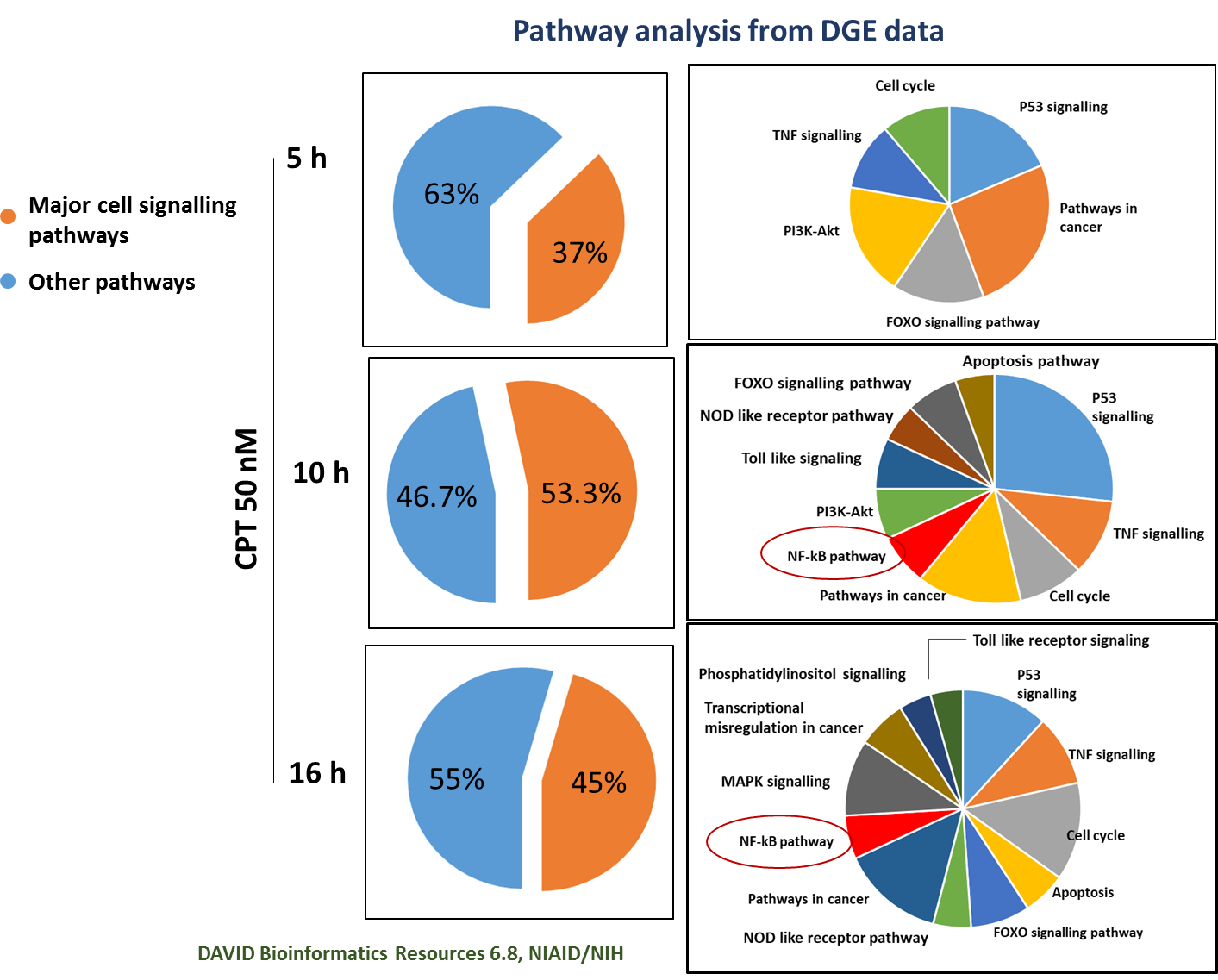


Supplementary Figure 2. Upregulated pathways in response to nanomolar concentration of CPT. Pathway analysis are carried out from the DGE analysis in high-throughput microarray data, obtained from cancer cells treated with 1 μM (5 h) and 50 nM (16 h) of CPT. NF-κB pathway upregulation were highlighted.


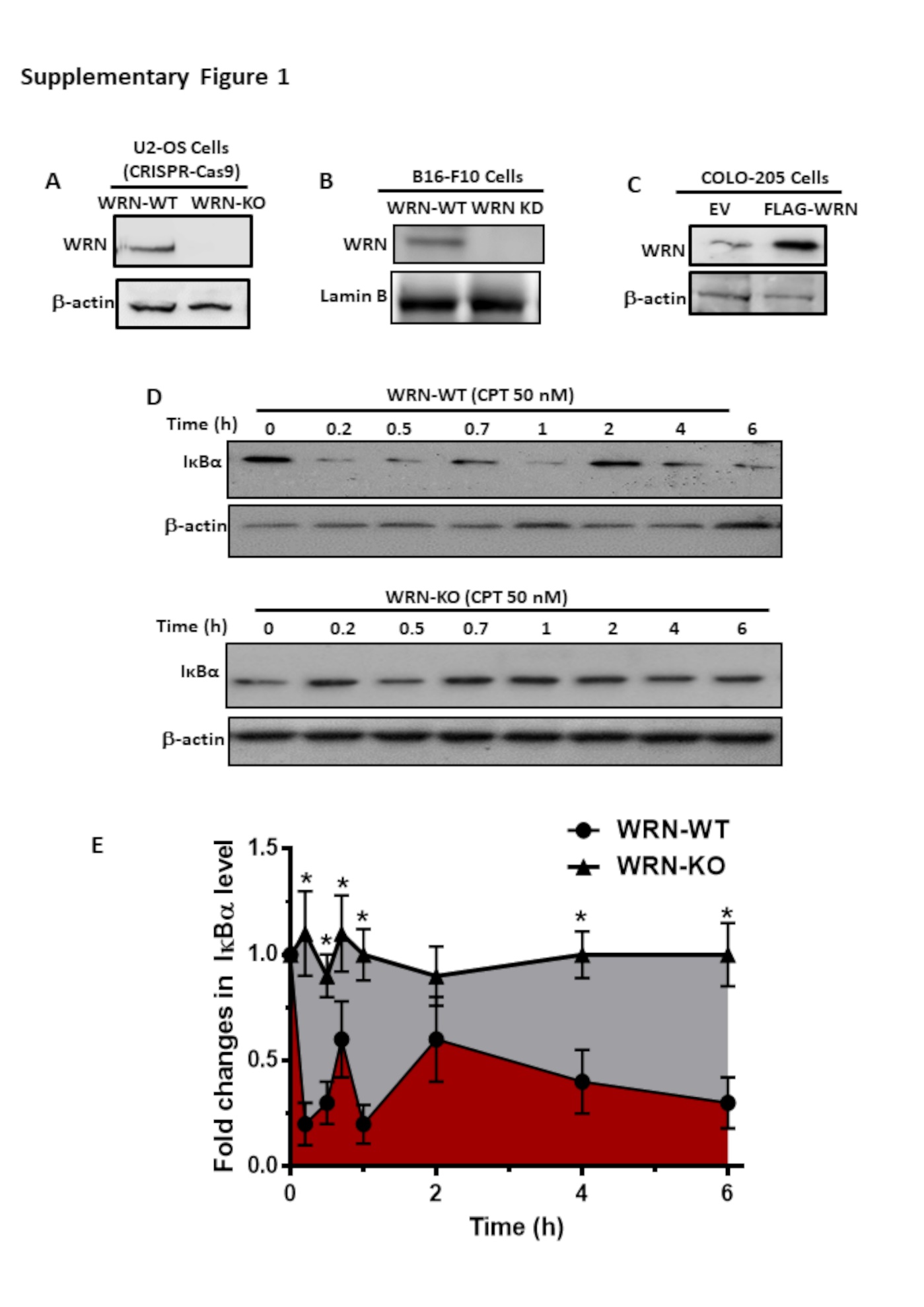


**Supplementary Figure 3. I**κ**Bα degradation is downregulated in WRN-deficient cells in response to TOP1cc. (A-C)** WRN knockout (WRN-KO) U2-OS cells were generated by CRISPR-CAS9 double nickase system. WRN-knockdown (WRN-KD) B16-F10 cells were generated by lentivirus mediated shRNA expression system. COLO205 cells were transfected with empty vector (EV) and FLAG-WRN. Expression of WRN in these cells were assessed by western blotting. **(D, E)** WRN-WT and WRN-KO U2-OS cells were treated with CPT (50 nM, 0-6 h) and IκBα level was assessed by western blotting and quantified. All the values indicated are mean ± S.E.M (n = 3). **p*<0.05 with respect to respective treatment in WRN-KO cells. *n.s.*: not significant. Bar in microscopic images: 5 μm

**
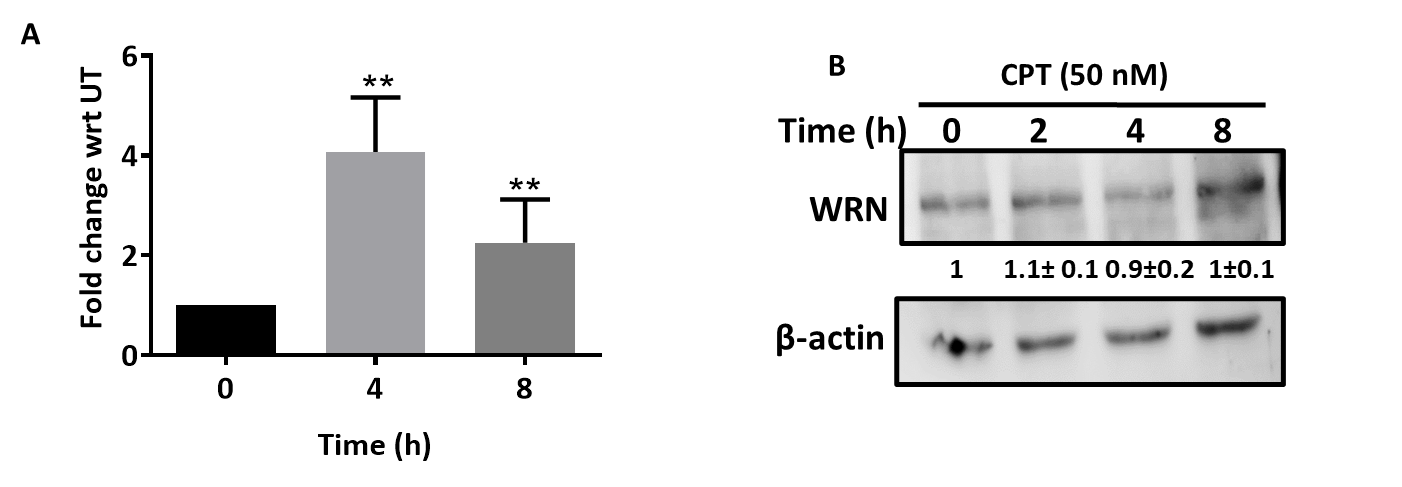
**

**Supplementary Figure 4. WRN mRNA level is enhanced while WRN protein level remains unaffected in response to nanomolar concentration of CPT. (A, B)** U2-OS cells were treated with CPT (50 nM, 0-8 h) and WRN mRNA and protein level were assessed by RT-PCR and western blot, respectively. All the values indicated are mean ± S.D (n = 3). ***p*<0.01 with respect to respective treatment at 0 h.

**
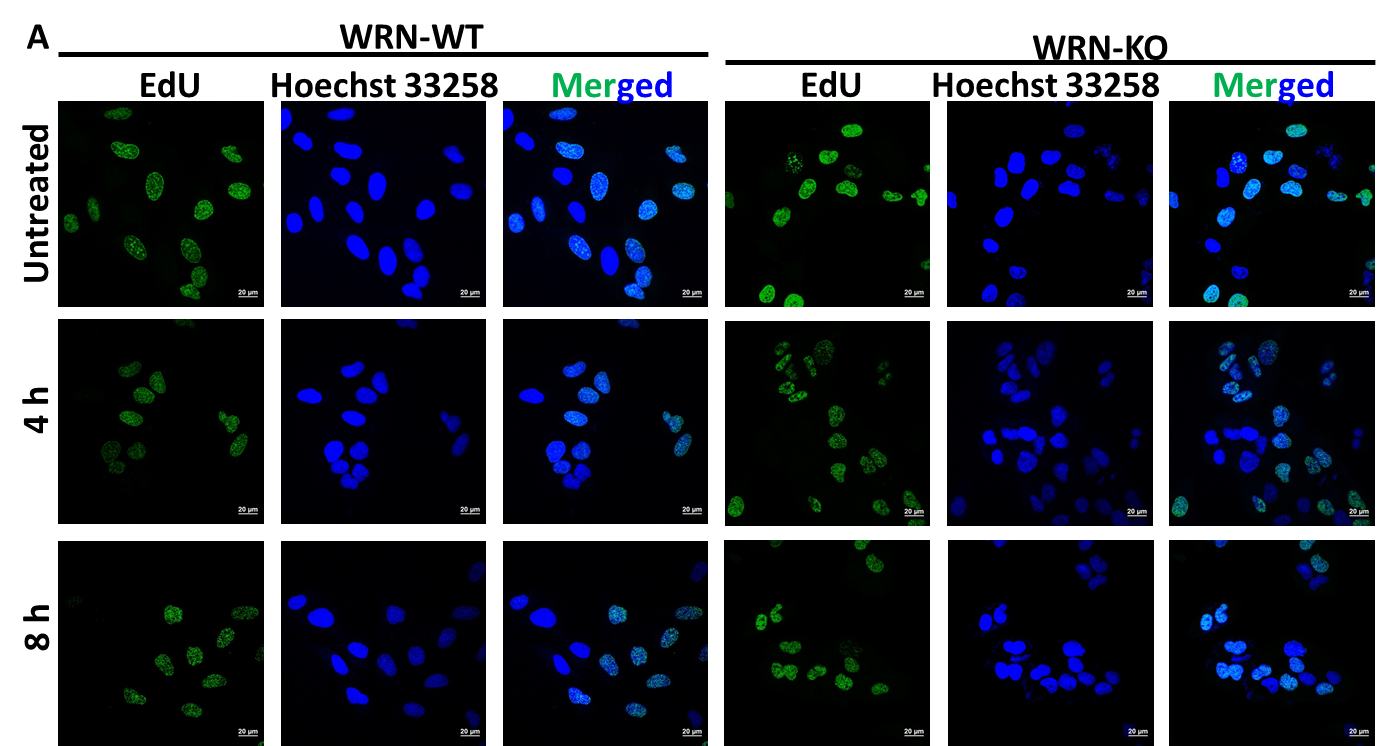
**

**
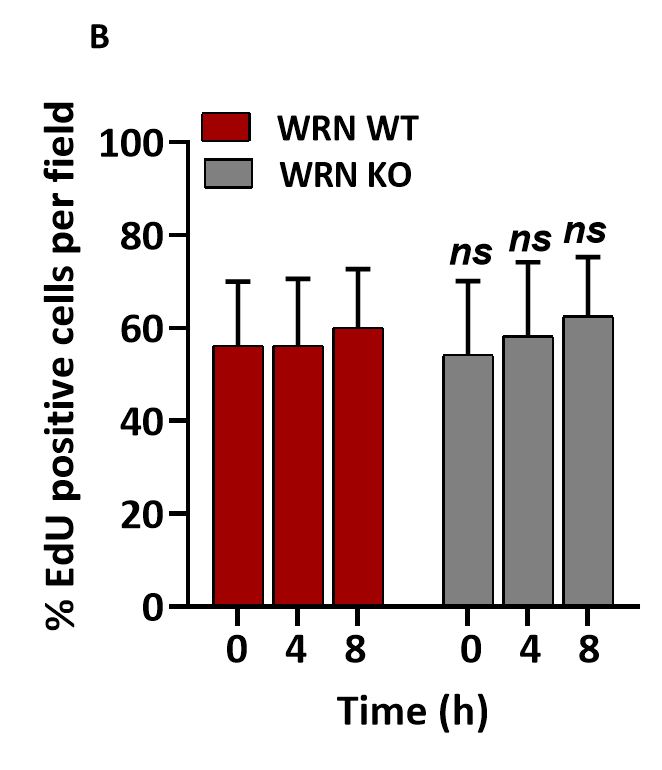
**

Supplementary Figure 5. Depletion of WRN does not affect the S-phase distribution in response to nanomolar concentration of CPT. (A, B) Cells were treated with CPT (50 nM) for indicated time periods and EdU incorporation was assessed by microscopy. All the values indicated are mean ± S.D (n = 3). *n.s.* indicates not significant.


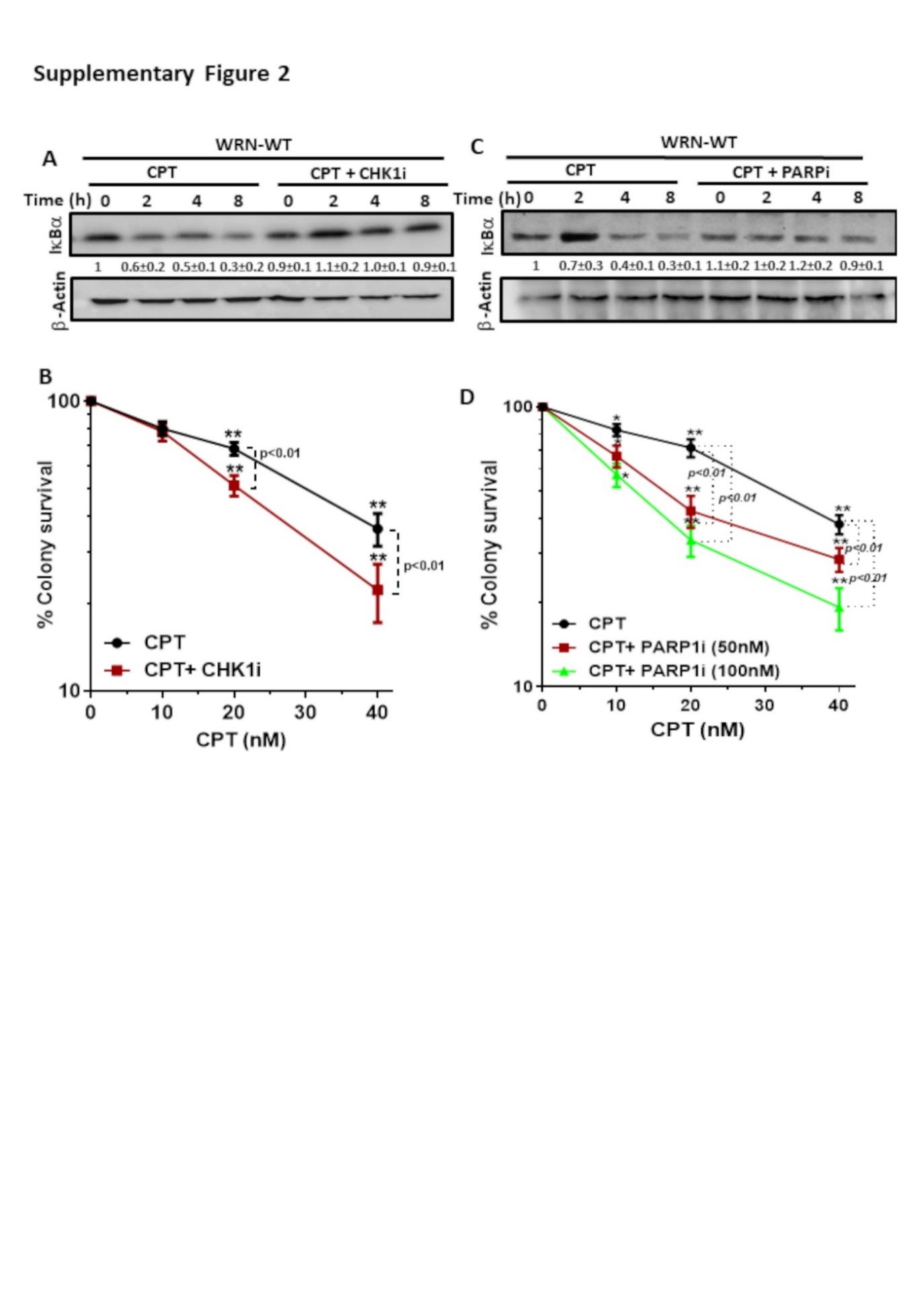


Supplementary Figure 6. WRN mediated NF-κB activation requires CHK1 and PARP1. (A) Cells were treated with CPT (50 nM) in the absence or presence of CHK1i (300 nM) for indicated time periods and NF-κB activation was assessed in term of IκBα degradation by western blotting. (B) WRN-WT U2-OS cells were treated with CPT, as mentioned above, in the absence or presence of CHK1 inhibitor (100 nM) and clonogenic survival was assessed. (C) Cells were treated with CPT (50 nM) in the absence or presence of PARPi (100 nM) for indicated time periods and NF-κB activation was assessed in term of IκBα degradation by western blotting. (D) WRN-WT U2-OS cells were treated with CPT, as mentioned above, in the absence or presence of PARP1 inhibitor (50 or 100 nM) and clonogenic survival was assessed. All the values indicated are mean ± S.D (n = 3 for A, C) or mean ± S.E.M (n = 6 for B, D). * *p*<0.05 or ***p*<0.01 with respect to vehicle treatment.


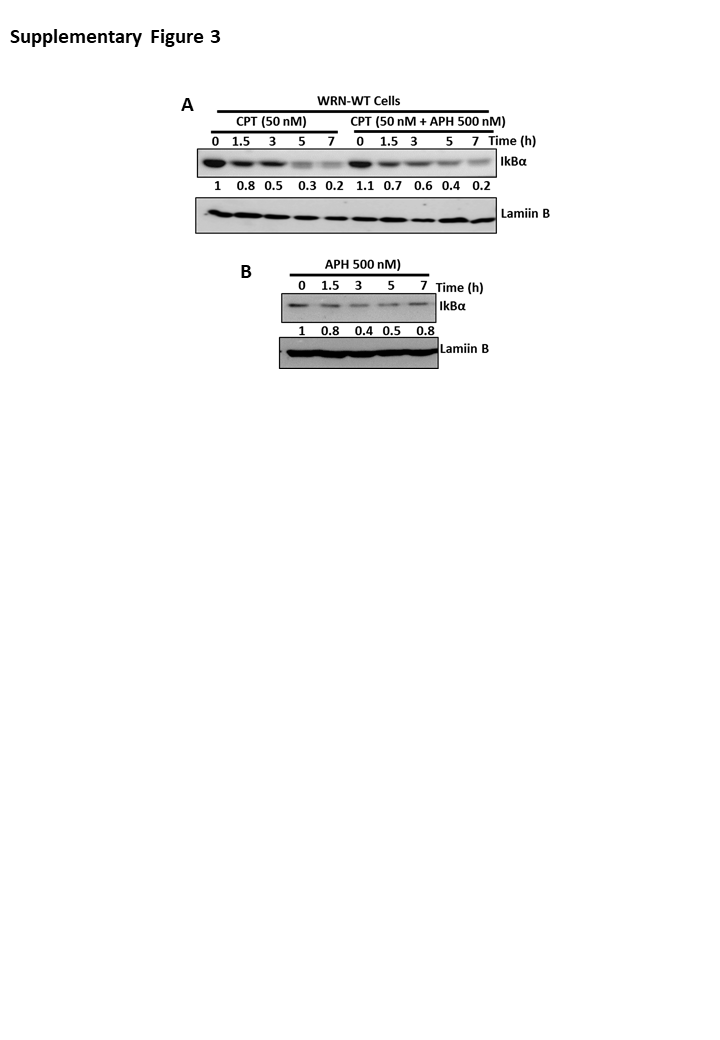


**Supplementary Figure 7. WRN regulated NF-**κ**B activation is independent of replication structures. (A, B)** WRN-WT cells were treated with CPT (50 nM) in the absence or presence of APH (500 nM) for indicated time periods and NF-κB activation was assessed in term of IκBα degradation by western blotting. For control, IκBα degradation was also assessed in cells treated with APH only.

**
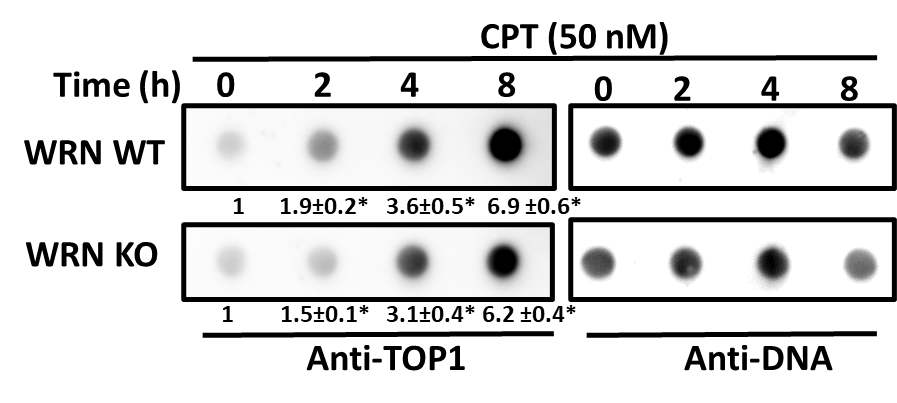
**

Supplementary Figure 8. TOP1cc removal is WRN independent in non-proliferating quiescent cells in response to nanomolar concentration of CPT. WRN-WT and KO cells were treated with CPT (50 nM) for indicated time periods and TOP1cc was assessed by RADAR based slot blot assay. All the values indicated are mean ± S.D (n = 4) **p*<0.05 with respect to respective 0 h treatment.


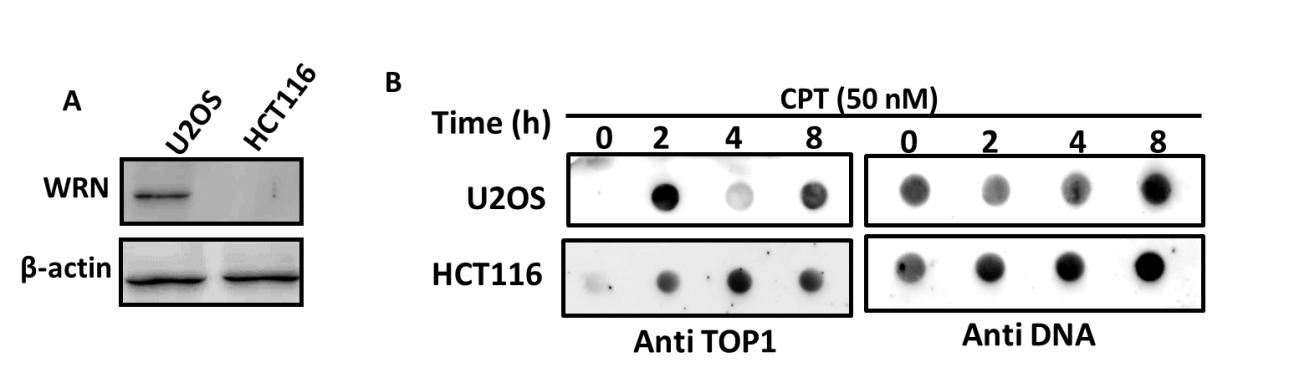


**
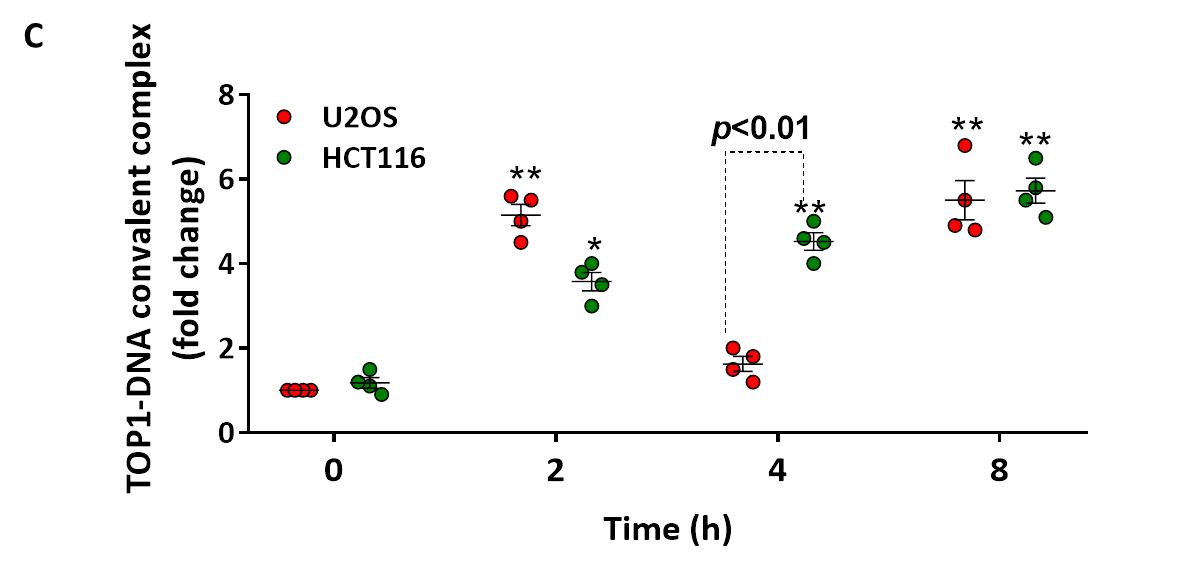
**

Supplementary Figure 9. WRN mediated TOP1cc removal is not cell line specific. (A) The level of WRN in U2-OS and HCT116 cells were assessed by western blotting. (B, C) U2-OS and HCT116 cells were treated with CPT (50 nM) for indicated time periods and TOP1cc was assessed by RADAR based slot blot assay. All the values indicated are mean ± S.E.M (n = 4) **p*<0.05, ***p*<0.01 with respect to respective 0 h treatment.


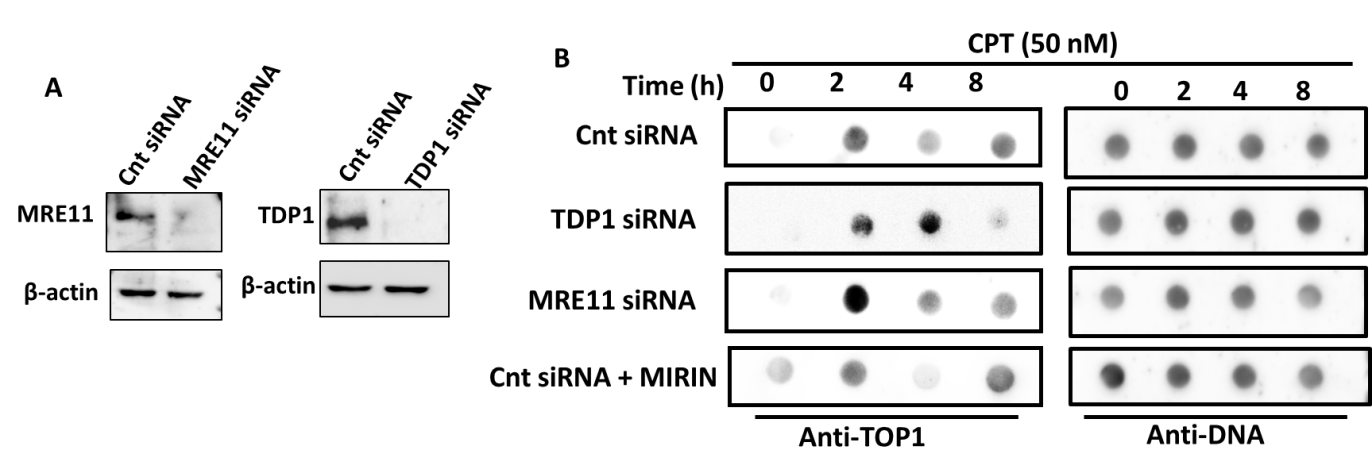


**
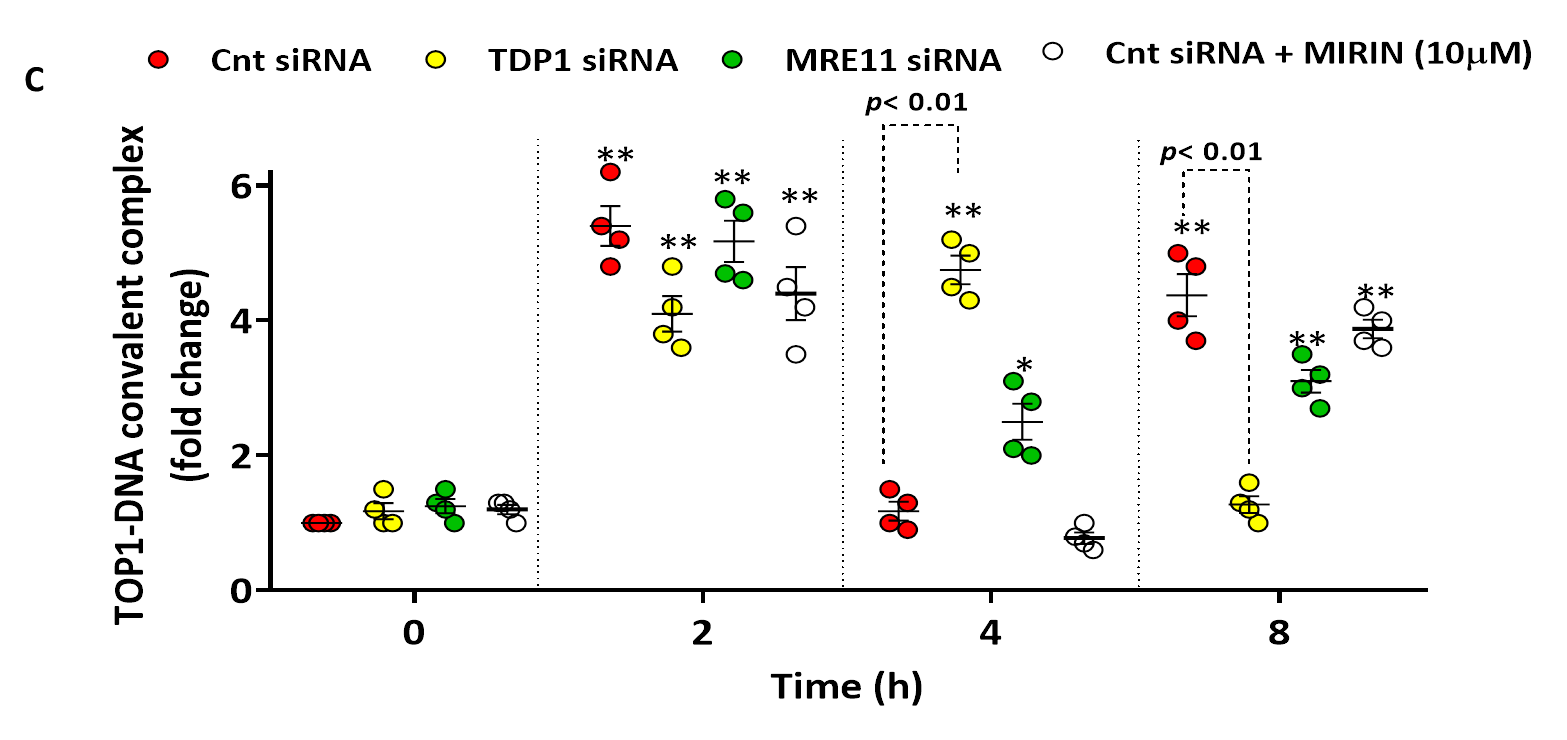
**

**Supplementary Figure 10. WRN mediated removal of TOP1cc is partially dependent on TDP1. (A)** U2-OS cells were treated with control, TDP1 or MRE11 siRNA for 24 h. and silencing of respective proteins were tested by western blotting. **(B, C)** Cells were treated with CPT (50 nM) in the absence or presence of mirin (10 μM) for indicated time periods and TOP1cc formation was assessed by RADAR based slot blot assay. DNA was probed as a loading control, in the same samples by DNA specific antibody, and all spots were quantified using densitometry. All the values indicated are mean ± S.E.M (n = 4). ***p*<0.01 with respect to respective cell types at 0 h.


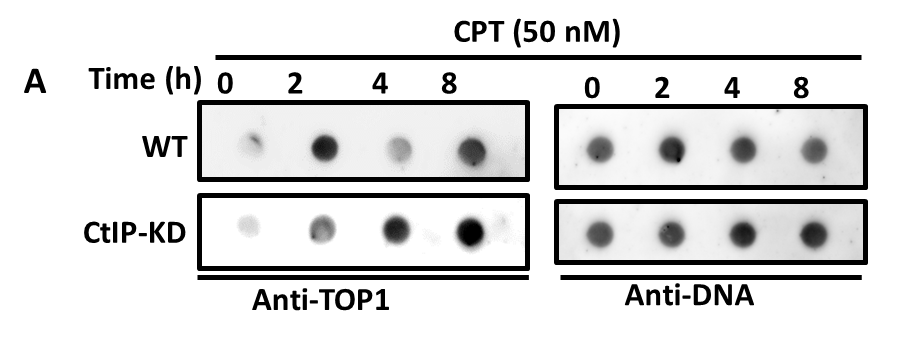


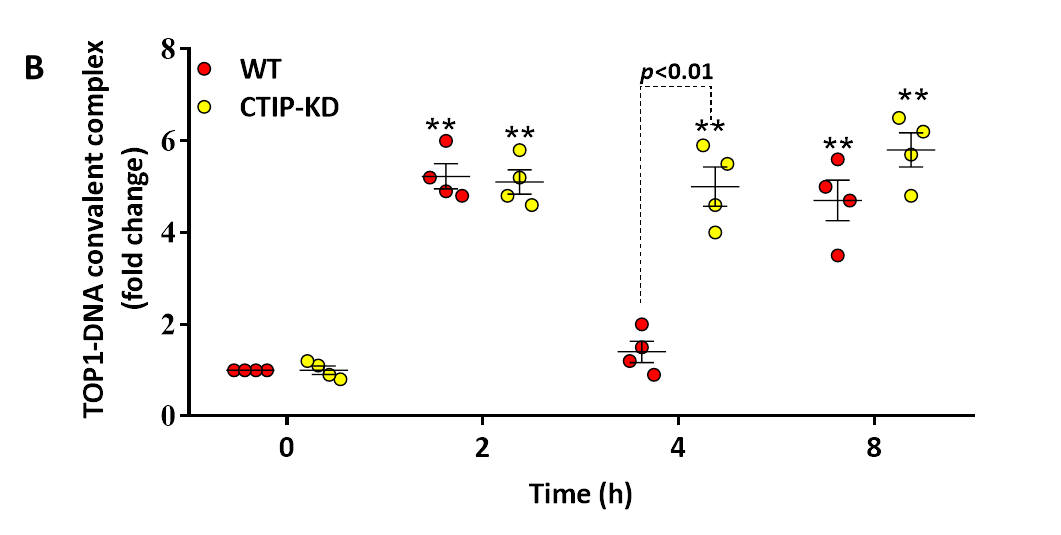


**Supplementary Figure 11. CTIP plays a significant role in WRN mediated removal of TOP1cc. (A, B)** WT (shRNA control) and CTIP-KD (CTIP shRNA) cells were treated with CPT (50 nM) for indicated time periods and TOP1cc formation was assessed by RADAR based slot blot assay. DNA was probed as a loading control, in the same samples by DNA specific antibody, and all spots were quantified using densitometry. All the values indicated are mean ± S.E.M (n = 4). ***p*<0.01 with respect to respective cell types at 0 h.


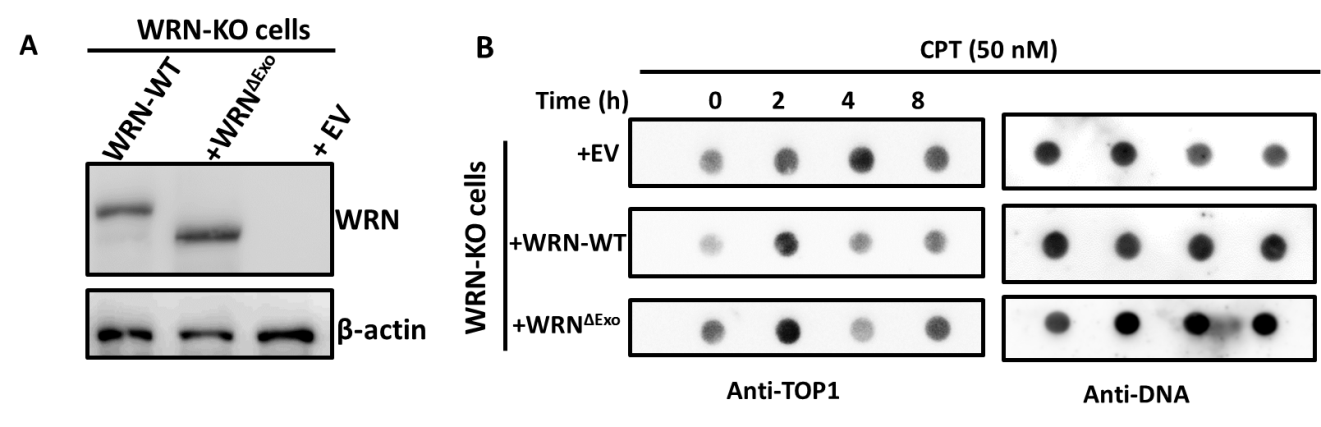


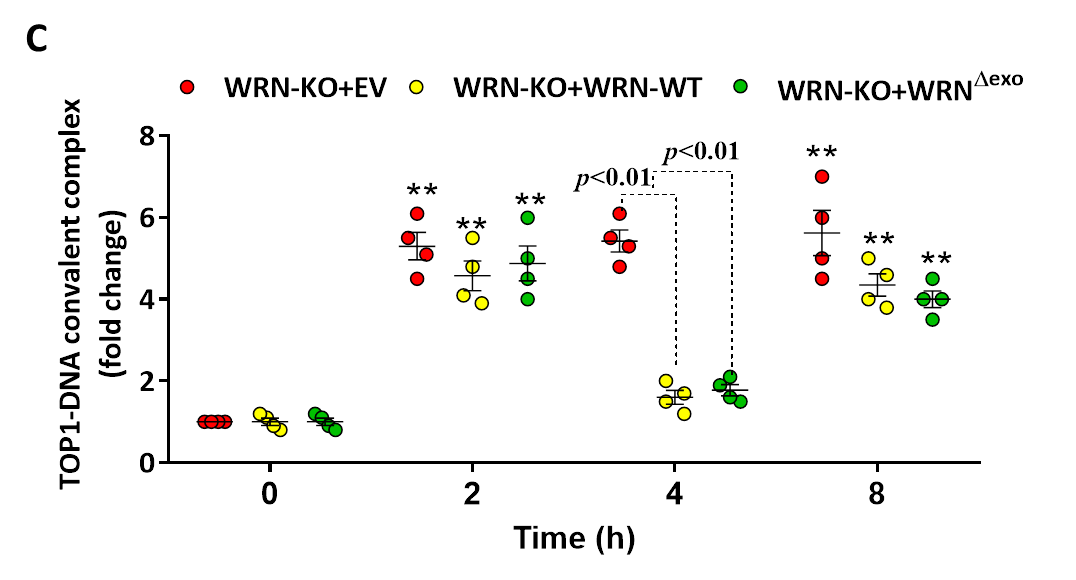


**Supplementary Figure 12. Nonenzymatic role of WRN in TOP1cc removal in U2-OS cells. (A)** WRN-KO cells were transfected for ectopic expression of EV (empty vector), WRN^WT^, and WRN**^Δ^**^EXO^. Expression level of WT and mutant WRN was assessed by western blotting. **(B, C)** WRN-KO cells were transfected for ectopic expression of EV (empty vector), WRN^WT^ and WRN**^Δ^**^EXO^. These cells were treated with CPT (50 nM) for indicated time periods and TOP1cc was analyzed by RADAR based slot blot assay. DNA was probed as a loading control, in the same samples by DNA specific antibody, and all spots were quantified using densitometry. All the values indicated are mean ± S.E.M (n = 4) ***p*<0.01 with respect to 0 h treatment in the respective cell types.


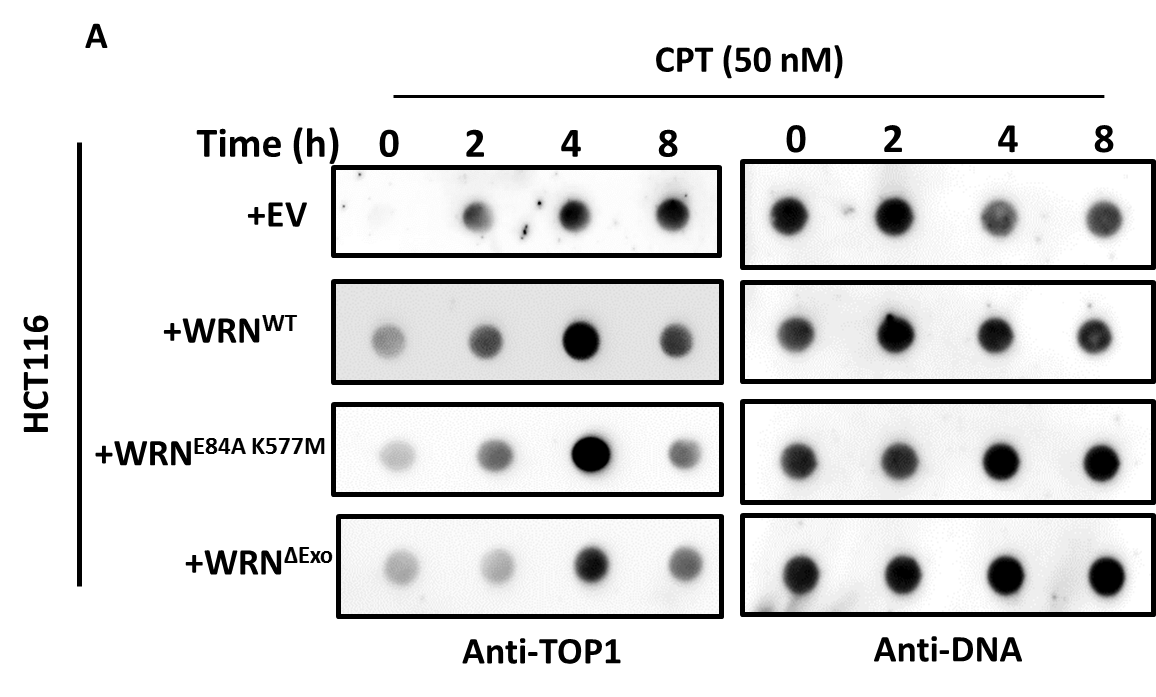


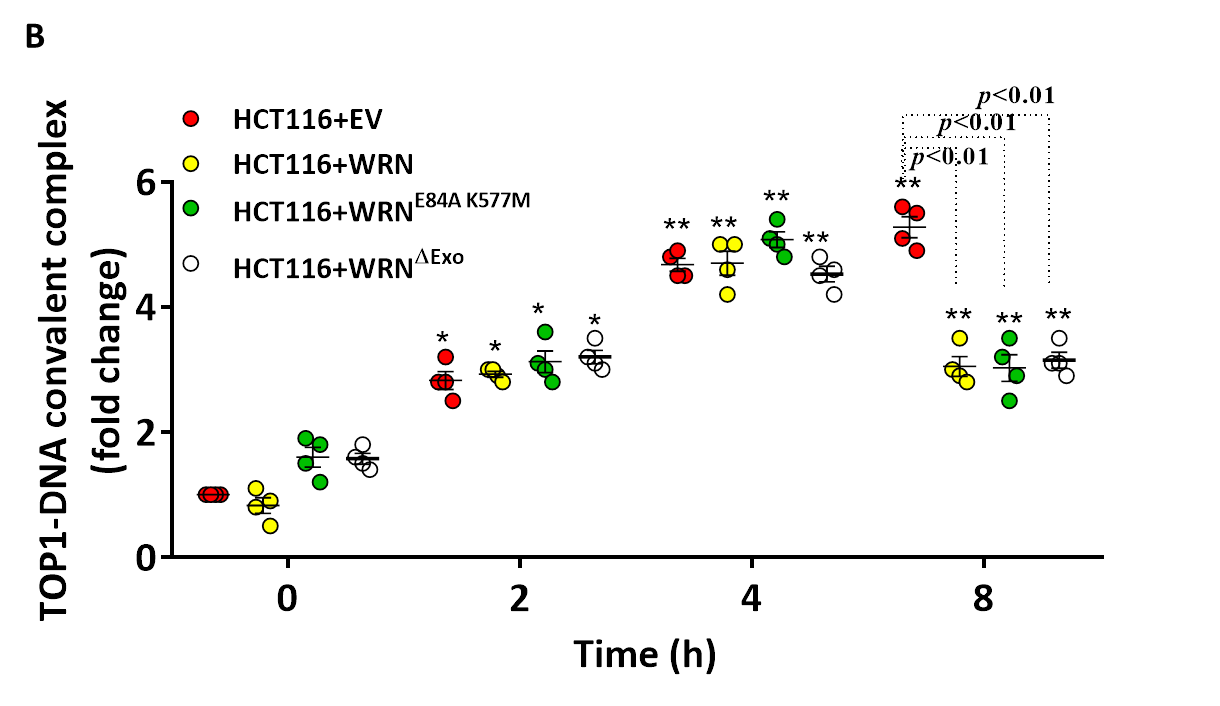


**Supplementary Figure 13. Nonenzymatic role of WRN in TOP1cc removal in HCT116 cells. (A, B)** HCT116 cells were transfected for ectopic expression of EV (empty vector), WRN^WT^, WRN^E84A-K577M^ or WRN**^Δ^**^EXO^. These cells were treated with CPT (50 nM) for indicated time periods and TOP1cc was analyzed by RADAR based slot blot assay. DNA was probed as a loading control, in the same samples by DNA specific antibody, and all spots were quantified using densitometry. All the values indicated are mean ± S.E.M (n = 4). *p*<0.05, ***p*<0.01 with respect to 0 h treatment in the respective cell types.


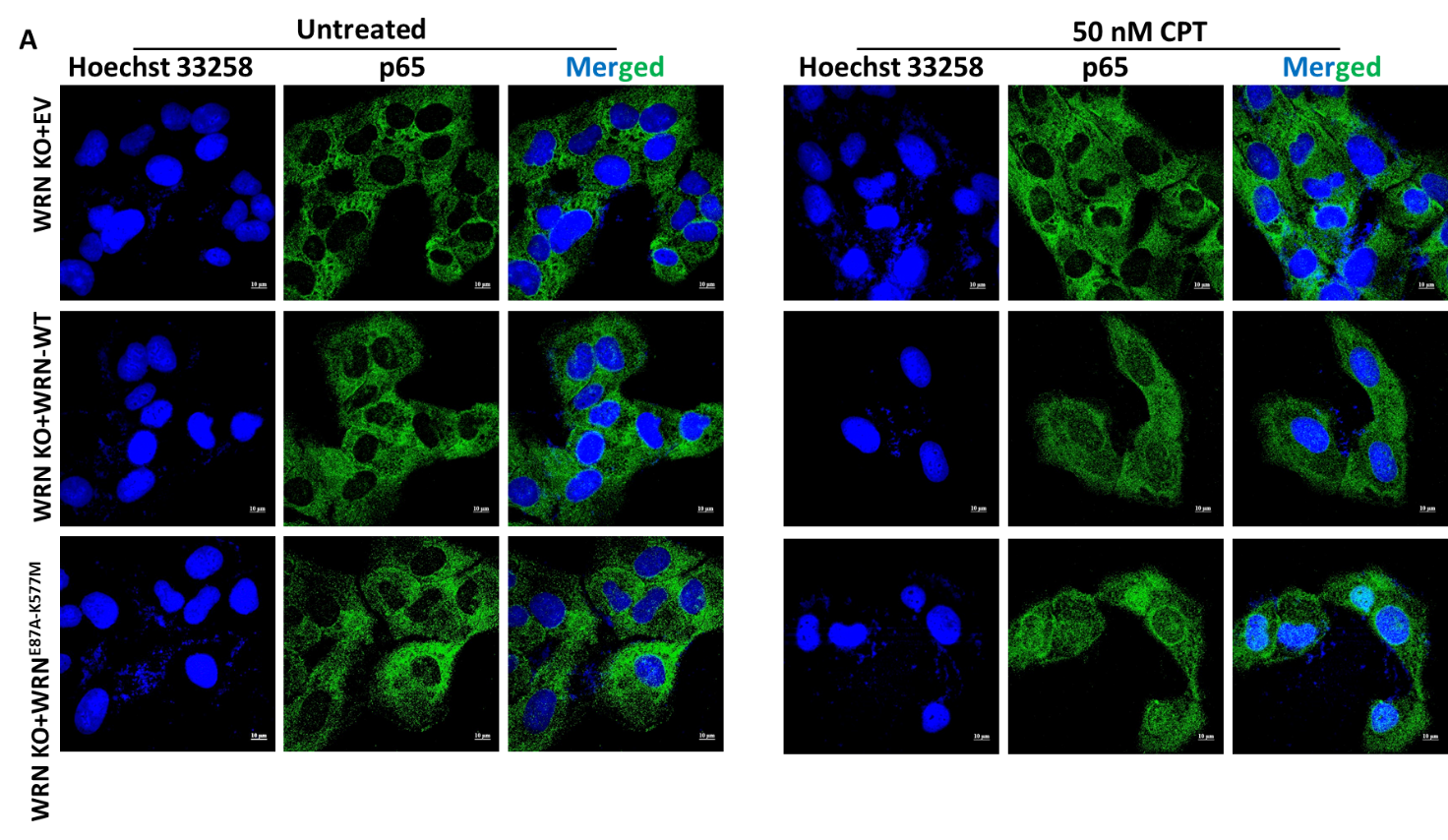


**B**


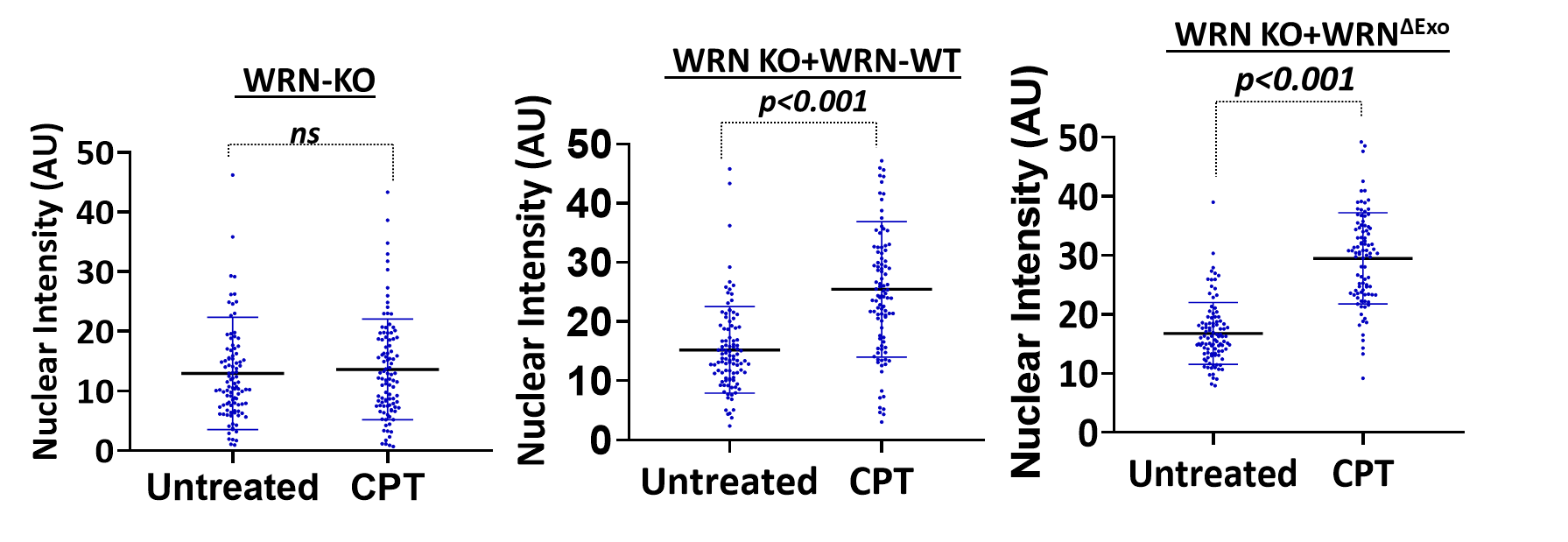


**Supplementary Figure 14. Nonenzymatic role of WRN in inducing nuclear localization of NF-kB in response to nanomolar concentration of CPT. (A, B)** WRN-KO cells were transfected for ectopic expression of EV (empty vector), WRN^WT^ or WRN**^ΔExo^**. These cells were treated with CPT (50 nM) for 8 h and the NF-κB activation was assessed in term of nuclear translocation of p65. Quantification of p65 intensity in the nucleus is shown in B. All the values indicated are mean ± S.E.M (n = 3-5). *n.s.*: not significant.


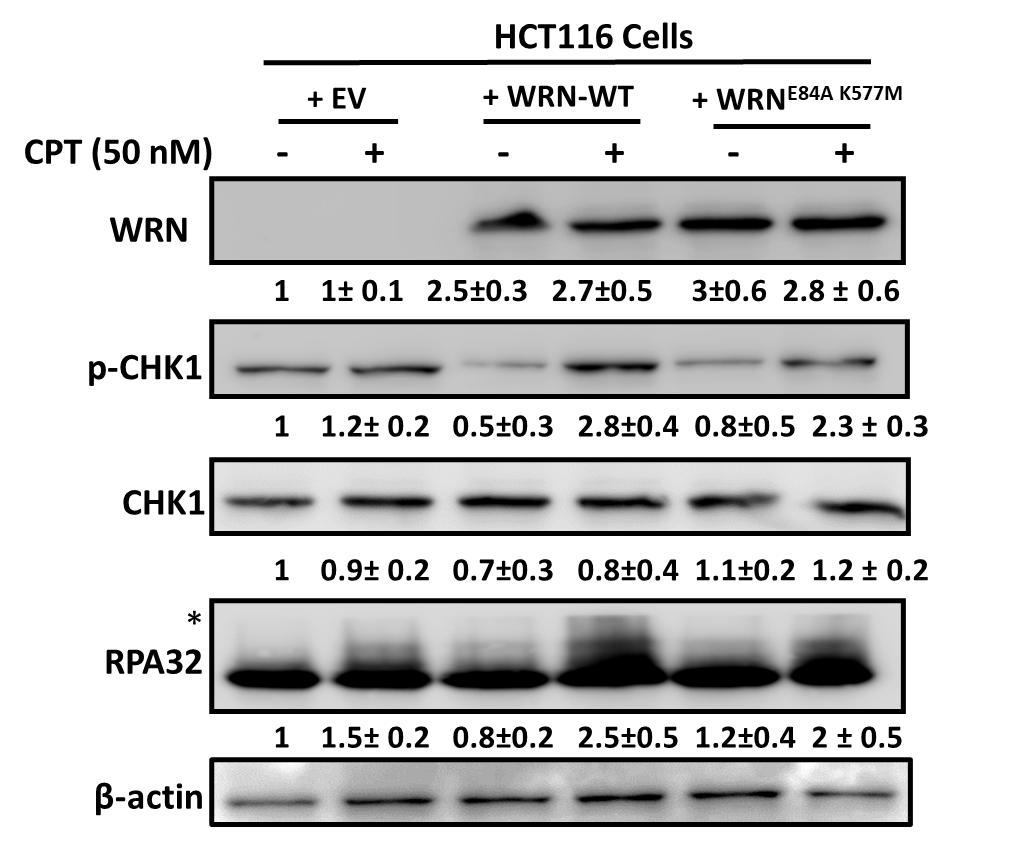


**Supplementary Figure 15. Nonenzymatic role of WRN in inducing CHK1 and RPA32 phosphorylation in HCT116 cells in response to nanomolar concentration of CPT.** HCT116 cells were transfected for ectopic expression of EV (empty vector), WRN^WT^ and WRN**^E84A-K577M^**. These cells were treated with CPT (50 nM) for 6 h and the levels of WRN, phosphorylation of CHK1 and RPA2 were assessed by western blotting. All the values indicated are mean ± S.D (n = 3). * represents slower migration of RPA2, owing to hyperphosphorylation.

**Supplementary Table 1. DGE for NF-kB related genes**

|  |
| --- |

|  |  | **Log (2) value for fold change in response to CPT treatment** | | | |
| --- | --- | --- | --- | --- | --- |
|  | **Gene symbol** | **50 nM 5 h** | **50 nM 10 h** | **50 nM 16 h** | **1 uM 5 h** |
| 1 | C1QTNF4 | 0.08747778 | 0.134404699 | 0.18288511 | 0.325117 |
| 2 | CALR | 0.04861648 | 0.010898543 | 0.1525012 | 0.1168 |
| 3 | CCL19 | 0.03668492 | 0.07098236 | -0.0874062 | 0 |
| 4 | CD14 | 0.1350637 | -0.244747653 | -0.1262651 | 0.179376 |
| 5 | CD86 | -0.0050068 | 0.138933575 | -0.0114078 | 0.021795 |
| 6 | CREBBP | -0.0352496 | -0.112011077 | 0.0653906 | -1.19822 |
| 7 | DDX3X | -0.1310949 | -0.23652164 | -0.2016592 | 0.018785 |
| 8 | EDA | -0.1063619 | 0.065013874 | -0.0403079 | 0.059645 |
| 9 | EDAR | 0.09295891 | 0.027873379 | 0 | 0.045143 |
| 10 | EDARADD\|ENO1P | -0.0311837 | 0.124142161 | 0.09599974 | 0.024823 |
| 11 | EDN1 | -0.10122 | -0.078628551 | 0.16857054 | 0.234278 |
| 12 | EGFR | -0.2715702 | -0.308416331 | -0.0555992 | -0.80973 |
| 13 | EIF2AK2 | -0.1478386 | 0.083103192 | -0.1614361 | -0.16101 |
| 14 | EP300 | -0.0284585 | -0.216236062 | 0.04026122 | -1.00921 |
| 15 | GLRX | -0.251441 | -0.140449514 | 0.05691919 | 0.116962 |
| 16 | GREM1 | 0.56714198 | 0.131229918 | 0.18698412 | 0.002761 |
| 17 | HAVCR2 | 0.09367564 | 0.061976882 | 0.04838561 | 0.616029 |
| 18 | HMGB1 | -0.0104532 | 0.016038407 | 0 | -0.01891 |
| 19 | IL18 | -0.1429758 | 0.076995737 | 0.1022558 | -0.35464 |
| 20 | IL18R1 | 0.03343498 | -0.017150412 | 0.01755438 | 0.144329 |
| 21 | IL1B | 0.04258439 | 1.067462912 | 0.78821763 | 0.633781 |
| 22 | ILK\|ILK-2 | -0.3245696 | -0.155435535 | 0.03578098 | -0.25457 |
| 23 | IRAK1 | -0.0688362 | 0.112271363 | -0.0924748 | 0.08287 |
| 24 | LIMS1 | -0.0144234 | -0.106260402 | -0.0843239 | -0.36144 |
| 25 | APP | 0.26561644 | -0.12405863 | -0.0609656 | 0.387005 |
| 26 | ACTN4 | 0.04826939 | -0.028140948 | -0.0203418 | -0.00683 |
| 27 | LRRC19 | 0 | 0.04602189 | -0.0242129 | -0.0189 |
| 28 | MMP8 | 0.11069384 | 0.138382906 | 0.07771498 | 0.015388 |
| 29 | NFAT5 | -0.0161858 | -0.168637286 | 0.03317826 | -1.16123 |
| 30 | NLRP12 | 0.15733157 | 0.09593009 | 0 | 0.025958 |
| 31 | NOD1 | 0.0304231 | 0.095702211 | 0.07411669 | 0.203681 |
| 32 | NOD2 | 0.00986954 | 0.078724863 | -0.0076617 | 0.284533 |
| 33 | NR3C2 | 0.0426005 | -0.061132353 | -0.0409279 | 0.054068 |
| 34 | PDCD4 | -0.064134 | 0.137797804 | 0.74952071 | 0.71904 |
| 35 | PHB2 | -0.0115024 | 0.04674612 | -0.053689 | 0.02553 |
| 36 | PTP4A3\|LOC100131062 | 0.128142 | 0.177499812 | 0.39722391 | 0.043545 |
| 37 | RBCK1 | 0.03040205 | 0.082236329 | 0.30255127 | 0.075568 |
| 38 | RC3H2 | 0.19644174 | 0.115792223 | 0.08802406 | 0.177944 |
| 39 | RELA | 0.02312474 | 0.14277454 | 0.15632335 | 0.015842 |
| 40 | RHOA | -0.3968839 | -0.169872294 | -0.1719742 | -0.19118 |
| 41 | RPS3 | 0.17592132 | 0.15738487 | 0.01140031 | 0.293296 |
| 42 | RTKN2 | -0.3553825 | -0.146798181 | -0.419889 | -0.47343 |
| 43 | SASH1 | 0.02776767 | 0.172705247 | 0.13346147 | -0.5122 |
| 44 | SMPD3 | -0.1224918 | -0.190486418 | -0.122313 | -0.271 |
| 45 | SPHK1 | 0.18632649 | 0.385074143 | -0.0384102 | 0.228924 |
| 46 | TERF2IP | 0.08749244 | 0.174192907 | 0.17777023 | 0.290297 |
| 47 | TLR2 | 0.10786861 | 0.244842012 | 0.06345517 | 0.282195 |
| 48 | TLR3 | 0.28184896 | 0.434542724 | 0.95199257 | 0.776437 |
| 49 | TLR4 | -0.0421476 | -0.106540755 | 0.08881135 | 0.060906 |
| 50 | TLR7 | 0.04217335 | 0.156907456 | 0.12337975 | 0.130434 |
| 51 | TLR9\|TWF2 | 0.15297229 | 0.206210976 | 0.24477188 | 0.246861 |
| 52 | TNFSF14 | -0.1611394 | 0.048387144 | 0.1405249 | 0.098625 |
| 53 | TRADD | 0.17309621 | 0.254628629 | 0.10711523 | 0.175596 |
| 54 | TRAF6 | 0.02265444 | -0.152871983 | 0.0092368 | 0.236141 |
| 55 | TREM2 | -0.0737251 | -0.090784613 | -0.1253037 | -0.00216 |
| 56 | TRIM44 | -0.0353579 | -0.036008952 | -0.0578679 | -0.27697 |
| 57 | TRIP6 | 0.09361088 | 0.201948209 | 0.58666138 | 0.05122 |
